# Planetary health diet index, genetic susceptibility and incident chronic kidney disease: a cohort study from the UK Biobank

**DOI:** 10.1016/j.jnha.2026.100777

**Published:** 2026-01-14

**Authors:** Duo Lv, Tingting Wang, Jiayao Fan, Dongsheng Hong, Zhiyi Chen, Qianchun Xu, Dan Zhou, Xishao Xie

**Affiliations:** a Department of Clinical Pharmacy, The First Affiliated Hospital, Zhejiang University School of Medicine. Hangzhou, China; b Department of Nutrition, The First Affiliated Hospital, School of Medicine, Zhejiang University, Hangzhou, China; c The Second Affiliated Hospital, School of Public Health, Zhejiang University School of Medicine, Hangzhou, China; d College of Medicine, Zhejiang University, Hangzhou, Zhejiang, China; e Department of Nephrology, Shulan (Hangzhou) Hospital, Zhejiang Shuren University, China; f Zhejiang University of Traditional Chinese Medicine, Hangzhou, Zhejiang, China; g Kidney Disease Center, The First Affiliated Hospital, Zhejiang University School of Medicine, Hangzhou, China

**Keywords:** Chronic kidney disease, Planetary health diet, Genetic susceptibility, UK Biobank

## Abstract

**Objective:**

The association between adherence to the planetary health diet and chronic kidney disease (CKD) remains under characterized. This study aim to investigate the association of planetary health diet index (PHDI) with the risk of CKD and assess potential effect modification by genetic predisposition.

**Design, setting, and participants:**

A large, population-based cohort study was conducted using data from UK Biobank. Eligible participants included those without a history of CKD who completed at least one 24 -h dietary recall questionnaire.

**Measurements:**

Three distinct planetary health diet indexes (PHDIs) were used to assess dietary adherence. A polygenic risk score (PRS) for CKD was calculated to evaluate genetic susceptibility. Cox proportional hazards models were used to estimate the associations between the PHDI and the risk of incident CKD. The joint effects of PHDI and genetic susceptibility were further examined. Sensitivity analyses were conducted to evaluate the robustness of the findings.

**Results:**

A total of 139,165 participants were included in the primary analysis. Over a median follow-up of 13.3 years, 6,391 incident CKD cases were identified. Compared with participants in the lowest adherence category, the hazard ratios (HRs) of incident CKD for those in highest adherence were 0.827 (95% CI, 0.757−0.904), 0.865 (95% CI, 0.805−0.929), and 0.891 (95% CI, 0.821−0.996) for Stubbendorff PHDI, Colizzi PHDI and Knuppel PHDI, respectively. Participants with highest adherence to planetary health diet and low genetic risk showed the lowest risk of CKD, with HRs of 0.707 (95% CI, 0.600−0.832), 0.682 (95% CI, 0.597−0.778), and 0.770 (95% CI, 0.663−0.893) across the three different PHDIs. These associations remained robust in several sensitivity analyses.

**Conclusions:**

Higher adherence to the planetary health diet was associated with lower risk of CKD, and these effects were enhanced by jointing with genetic susceptibility. Promoting this sustainable dietary pattern may play a key strategy for CKD prevention.

## Graphical abstract

**Figure:**
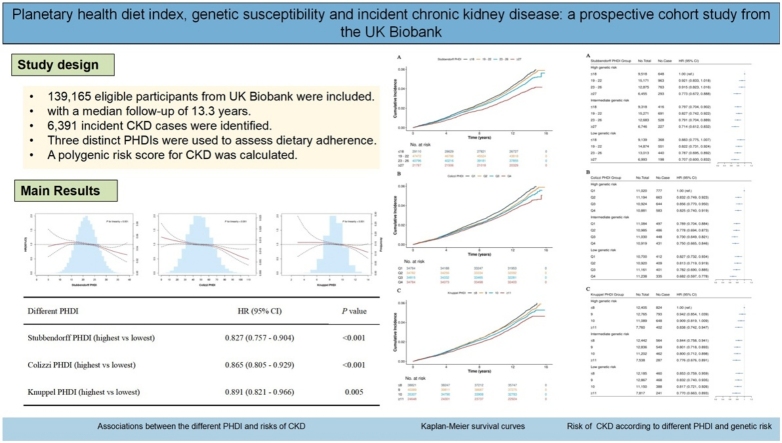

## 1 Introduction

Chronic kidney disease (CKD) is a significant global health concern and a major contributor to morbidity and mortality worldwide. Approximately 10% of adults globally are affected by CKD, accounting for 1.2 million deaths and 28.0 million years of life lost annually [1,2]. Projections suggest that CKD will become the fifth leading cause of death worldwide by 2040 [3]. The insidious nature of CKD often renders affected individuals asymptomatic until advanced stages of the disease. Given the profound impact and substantial healthcare burden of CKD, identifying modifiable risk factors for its primary prevention is an urgent public health priority.

Poor-quality dietary habits are among the critical risk factors for the onset and progression of CKD. Dietary modifications are widely recommended as a key non-pharmacological strategy for both the prevention and management of CKD [4]. Current evidence indicates that restricting dietary protein intake and adopting a plant-based diet may reduce the risk of CKD progression and improve biochemical outcomes [5]. However, much of the existing research has largely focused on the effects of individual nutrients or specific food groups on CKD progression. Exploring comprehensive dietary structures, which encompass a broader combination of nutrients and food consumption, may provide deeper insights into their preventive effects on CKD incidence. Although previous studies have explored the relationship between various dietary patterns and CKD risk, these patterns have primarily focused on human health while neglecting the environmental impact of food production and its implications for sustainable development. In 2019, the EAT-Lancet Commission proposed a global reference diet known as the planetary health diet, which aims to promote human health while ensuring environmental sustainability [6]. The planetary health diet emphasizes a predominant consumption of vegetables, fruits, legumes, nuts and whole grains, moderate intake of fish, and a low consumption of meat, dairy products, tubers, refined cereals, added fats, and sugars. The EAT-Lancet Commission highlights that the planetary health diet provides a more comprehensive approach to addressing dietary and agricultural components of environmental change compared to most national food-based dietary guidelines [7]. Since its introduction, several studies have demonstrated associations between adherence to the planetary health diet and reduced risks of multiple diet-related chronic diseases and mortality [[8], [9], [10], [11], [12]]. However, the relationship between the planetary health diet and CKD risk remains unclear. Additionally, genetic factors have emerged as significant contributors to CKD risk in recent years. Genome-wide association studies (GWASs) have identified numerous genetic loci associated with kidney function, which are robustly linked to estimated glomerular filtration rate (eGFR) and the risk of adverse kidney outcomes [[13], [14], [15], [16]]. It remains uncertain whether genetic susceptibility modifies the association between adherence to the planetary health diet and the risk of incident CKD.

Thus, the primary objective of this study was to investigate the associations between adherence to the planetary health diet and the incidence of CKD in the UK Biobank population. A secondary objective was to examine whether these associations were modified by the genetic susceptibility of CKD.

## 2 Methods

### 2.1 Study population

The UK Biobank is a large, population-based prospective cohort that recruited over 500,000 participants aged 37–73 years across the United Kingdom between 2006 and 2010. The UK Biobank study was approved by the North West Multi-center Research Ethics Committee (21/NW/0157), and all participants provided informed consent. Health information was collected through touchscreen questionnaires, face-to-face interviews, physical measurements, and biological sample collection at 22 assessment centers in Scotland, England, and Wales. Participants were followed up using linkages to national registers for incident diseases, hospital admissions, and deaths.

**Fig. 1:**
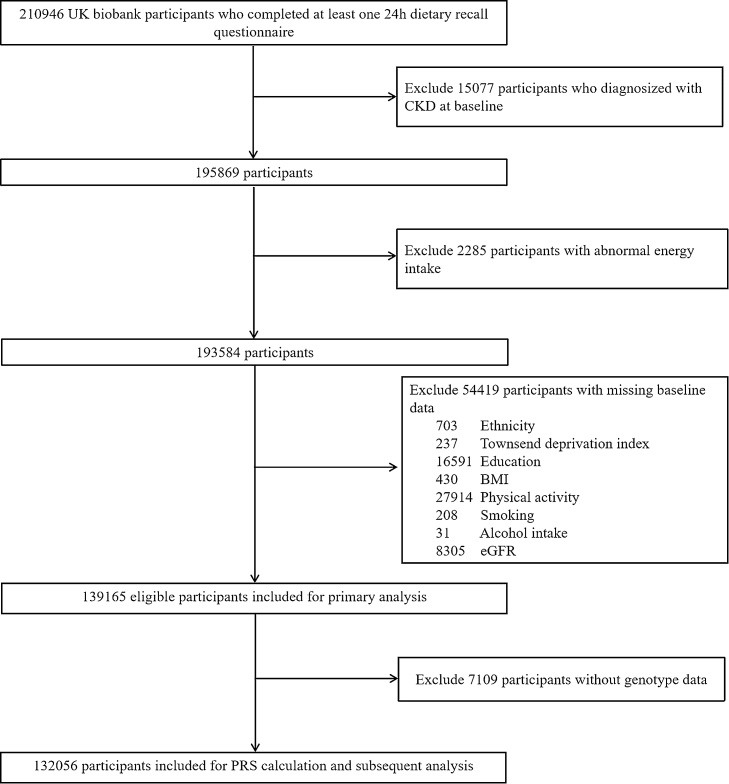
Study Participant Flow. Abbreviations: CKD, chronic kidney disease; BMI, body mass index; eGFR, estimated glomerular filtration rate; PRS, polygenic risk score

Participants who had completed the online 24-h dietary recall questionnaire on at least one occasion were eligible for inclusion. Exclusions included individuals with a pre-baseline diagnosis of CKD, missing eGFR data or dietary intake information, and those reporting abnormal energy intakes (<800 or >4200 kcal/day for men and <500 or >3500 kcal/day for women). The flowchart of the study population was shown in Fig. 1.

### 2.2 Dietary assessment and planetary health diet index

Dietary information was collected using the Oxford WebQ, a web-based self-administered 24 -h dietary questionnaire. Participants completed this questionnaire during their baseline assessment (2009–2010) and were invited to complete four additional online questionnaires between February 2011 and April 2012. A total of 210,946 participants completed at least one dietary assessment, either online or at baseline. The concrete weight of each consumed food used for diet index calculations was collected [17] (https://biobank.ndph.ox.ac.uk/showcase/label.cgi?id=100118). For participants who completed multiple dietary assessments, average intakes of each food item were calculated.

The planetary health diet index (PHDI) was used to assess adherence to the EAT-Lancet Commission's recommendations on healthy diets from sustainable food systems. While several approaches have been developed to translate the planetary health diet into dietary scores, no consensus exists on quantification methods. A Previous study [18] has systematically compared seven different PHDIs on health and environmental outcomes, and identified the Stubbendorff PHDI and Colizzi PDHI as the most consistent in grouping participants and relating to disease and climate outcomes. across the validation cohorts. Therefore, the Stubbendorff and Colizzi PHDI were adopted in our study. Additionally, the Knuppel PHDI, which was the first proposed and most widely used index, was also performed to assess the robustness of the relationship between the relationship between planetary health diet adherence and incident CKD and incident CKD. Each PHDI uses a different scoring system: the Stubbendorff PHDI employs an ordinal scale, the Colizzi PHDI uses a proportional scoring system, and the Knuppel PHDI uses a binary system. Details of these three PHDIs have been described in their development studies [[19], [20], [21]] and Table S1-S5.

### 2.3 Identification of CKD

Incident CKD was identified through primary care data, hospital inpatient records, and death registry entries, based on ICD and OPCS4 codes (Table S6). Baseline CKD was defined as eGFR less than 60 mL/min per 1.73m^2^ (calculated using the CKD-EPI equation) or albuminuria exceeding 30 mg/L at recruitment, or cases identified through ICD/OPCS4 codes above mentioned. The follow-up period was defined as the time from the date of attendance at the assessment center to the date of first CKD diagnosis, death, loss to follow-up, or the end of the follow-up period (October 22, 2022, for England; August 31, 2022, for Scotland; and May 31, 2022, for Wales), whichever occurred first.

### 2.4 Covariates assessment

The following covariates, including sociodemographic characteristics and lifestyle factors, were collected at baseline: age, sex, education level, body mass index (BMI, categorized as <18.5, 18.5−24.9, 25−29.9, or ≥ 30 kg/m²), Townsend deprivation index (reflecting area-level socioeconomic status, based on participants’ residential postcode), smoking status (classified as never, former and current smoking), alcohol intake (categorized into 6 levels based on frequency), physical activity (low: <600 MET min/week, moderate: 600–3000 MET min/week, high: ≥3000 MET min/week, based on the International Physical Activity Questionnaire [IPAQ]). Prevalent hypertension, diabetes, and cardiovascular disease (CVD) were also recorded at baseline based on ICD and OPCS4 codes (Table S7). Total energy intake was assessed by the 24 -h dietary recall questionnaire.

### 2.5 Genetic risk assessment

To calculate the polygenic risk score (PRS) for CKD, we utilized imputed genotype data from the UK Biobank (https://biobank.ndph.ox.ac.uk/showcase/label.cgi?id=263). Genotyping methods and imputation details have been previously reported [22]. Quality control (QC) was performed on the genotype data, retaining only single nucleotide polymorphisms (SNPs) with a minor allele frequency (MAF) greater than 0.01 and passing Hardy-Weinberg equilibrium test with a *p*-value threshold of 1e-6. From a prior GWAS that identified a total of 40,042 SNPs associated with renal function [13], we successfully mapped 38,508 SNPs to the post-QC UK Biobank dataset (Supplementary File 2). These SNPs were finally used to construct PRS using a weighted approach. Specifically, each SNP was coded as 0, 1, or 2 based on the number of effect alleles present. The PRS was then computed as the sum of the product of these coded counts and their corresponding effect sizes derived from the GWAS. Participants with higher PRS values exhibited better kidney function and a lower likelihood of developing CKD. Therefore, participants were categorized into three genetic risk groups: "low genetic risk," "intermediate genetic risk," and "high genetic risk," based on the tertiles of the PRS, organized from highest to lowest.

### 2.6 Statistical analysis

The baseline demographic, biochemical and clinical characteristics were presented using means with standard deviations (SDs) or medians with interquartile range (IQR) for continuous variables, and as frequencies with percentages for categorical variables. Penalized splines [23,24] were performed to explore the nonlinear associations of these three PHDIs with incident CKD (Methods S1). In Cox models with penalized splines, the linear-response associations of three different PHDIs with incident CKD risk were presented after adjustment for confounding factors (Figure S1). According to the Cox models with penalized splines and to ensure adequate number of participants in each group, the participants were divided into four groups for the Stubbendorff PHDI (≤18, 19−22, 23−26, and ≥27 points) and Knuppel PHDI (≤8, =9, =10, ≥11 points), respectively. Participants were divided into quartiles according to the Colizzi PHDI. The cumulative probabilities of incident CKD stratified by the different PHDIs were performed using the Kaplan-Meier method. Cox proportional hazards models were used to estimate the hazard ratios (HRs) with 95% confidence intervals (CIs) for the associations between the different PHDI and the risk of incident CKD. The lowest group was used as the reference, and three adjustment models were analyzed. Model 1 was adjusted for age, sex and ethnicity; model 2 was adjusted for model 1 plus Townsend index, educational level and baseline comorbidities (including hypertension, diabetes, cardiovascular disease, and cancer); and model 3 was adjusted for model 2 plus BMI, eGFR, smoking status, alcohol intake, physical activity and total energy intake. To investigate whether the association of PHDIs with incident CKD was modified by genetic susceptibility, additional Cox proportional hazards models was performed according to the tertiles of the PRS. The joint effects of PHDI and genetic susceptibility were further examined, and the group of lowest adherence to planetary health diet and high genetic risk was considered as the reference.

Several sensitivity and subgroup analyses were conducted to evaluate the robustness of our findings. First, we employed competing risk models proposed by Fine and Gray [25] to minimize the competitive risk bias caused by death, enabling a more accurate assessment of the impact of PHDI on incident CKD. Second, we excluded participants diagnosed with CKD within the first three years of follow-up to reduce the potential for reverse causality. Third, we repeated the analysis using the follow-up phase which began at the time when the latest dietary questionnaire was completed. Fourth, we excluded participants that completed only one 24-h dietary recall questionnaire. Fifth, mediation analyses were conducted to quantify the proportion of CKD risk attributable to indirect factors, with mediation proportions (95% CIs) calculated for BMI, hypertension, diabetes, and cardiovascular disease. Sixth, stratified subgroup analyses were also performed to investigate the potential interaction effects by adding interaction terms between the PHDIs and covariates in the model. Seventh, a different PRS calculation method based on SNPs associated with renal function reported by Yu et al. [14] was used to repeat the gene association analysis to minimize potential biases arising from the limitations of specific algorithms (Supplementary File 2). Finally, we also evaluated the associations of between PHDI and incident CKD after imputing individuals with missing baseline covariates using multiple imputation methods (Methods S2).

All statistical analyses were conducted using R software (version 4.1.1). All statistical tests were two-sided, and *P* values < 0.05 were considered statistically significant.

## 3 Results

### 3.1 Baseline characteristics

A total of 139,165 participants were included in the study, and the baseline characteristics of the overall cohort are presented in Table 1. The median age of the participants was 56.0 (IQR, 42.0–62.0) years, and 46.7% were men. The median Stubbendorff PHDI was 22 (range, 5–39). According to categories of the Stubbendorff PHDI, Participants with higher Stubbendorff PHDI scores were more likely to be female, highly educated, non-smokers, physically active, and have lower BMI and total energy intake. They also had a lower prevalence of diabetes, cardiovascular disease (CVD), and hypertension (Table 1). The median Colizzi PHDI and Knuppel PHDI was 47 (range, 0–111) and 9 (range, 3–14), respectively. Baseline characteristics according to the Colizzi PHDI or Knuppel PHDI were presented in Table S8-S9.

**Table 1:** Baseline characteristics of the study participants according to categories of the Stubbendorff PHDI.

Characteristics	Total	Categories of the Stubbendorff PHDI			
		≤18	19−22	23−26	≥27
No. participants	139165	29110	47472	40796	21787
Age (years)	56.0 (49.0, 62.0)	55.0 (48.0, 61.0)	56.0 (49.0, 62.0)	56.0 (49.0, 62.0)	56.0 (49.0, 62.0)
Sex (male, %)	65058 (46.7)	16420 (56.4)	22992 (48.4)	17396 (42.6)	8250 (37.9)
Ethnicity (%)					
White	133677 (96.1)	28332 (97.3)	45874 (96.6)	39025 (95.7)	20446 (93.8)
Asian	1489 (1.1)	257 (0.9)	490 (1.0)	463 (1.1)	279 (1.3)
Black	2195 (1.6)	241 (0.8)	527 (1.1)	769 (1.9)	658 (3.0)
Other	1804 (1.3)	280 (1.0)	581 (1.2)	539 (1.3)	404 (1.9)
Education (%)					
College or University degree	68168 (49.0)	12474 (42.9)	22405 (47.2)	20954 (51.4)	12335 (56.6)
Other	70997 (51.0)	16636 (57.1)	25067 (52.8)	19842 (48.6)	9452 (43.4)
Townsend deprivation index	−2.4 (−3.8, −0.1)	−2.4 (−3.8, −0.2)	−2.5 (−3.8, −0.3)	−2.4 (−3.8, 0.0)	−2.1 (−3.7, 0.4)
BMI (%, kg/m2)					
Mean (SD)	26.6 (4.4)	27.4 (4.5)	26.9 (4.4)	26.4 (4.4)	25.6 (4.2)
<18.5	730 (0.5)	105 (0.4)	196 (0.4)	224 (0.5)	205 (0.9)
18.5−24.9	54203 (38.9)	9400 (32.3)	17245 (36.3)	16840 (41.3)	10718 (49.2)
25−29.9	58069 (41.7)	12854 (44.2)	20509 (43.2)	16739 (41.0)	7967 (36.6)
≥30	26163 (18.8)	6751 (23.2)	9522 (20.1)	6993 (17.1)	2897 (13.3)
Smoking (n, %)					
Never	80439 (57.8)	16180 (55.6)	27281 (57.5)	24029 (58.9)	12949 (59.4)
Previous	48296 (34.7)	9896 (34.0)	16540 (34.8)	14195 (34.8)	7665 (35.2)
Current	10430 (7.5)	3034 (10.4)	3651 (7.7)	2572 (6.3)	1173 (5.4)
Alcohol intake (%)					
Never	7644 (5.5)	1362 (4.7)	2309 (4.9)	2383 (5.8)	1590 (7.3)
Special occasions only	11979 (8.6)	2235 (7.7)	3948 (8.3)	3622 (8.9)	2174 (10.0)
1−3 times/month	14771 (10.6)	3039 (10.4)	4872 (10.3)	4381 (10.7)	2479 (11.4)
1−2 times/week	34294 (24.6)	7049 (24.2)	11777 (24.8)	10131 (24.8)	5337 (24.5)
3−4 times/week	36856 (26.5)	7566 (26.0)	12751 (26.9)	10846 (26.6)	5693 (26.1)
Daily or almost daily	33621 (24.2)	7859 (27.0)	11815 (24.9)	9433 (23.1)	4514 (20.7)
Physical activity (%)					
Low	24808 (17.8)	6434 (22.1)	8793 (18.5)	6668 (16.3)	2913 (13.4)
Moderate	59479 (42.7)	12404 (42.6)	20668 (43.5)	17339 (42.5)	9068 (41.6)
High	54878 (39.4)	10272 (35.3)	18011 (37.9)	16789 (41.2)	9806 (45.0)
Cancer (%)	14135 (10.2)	2817 (9.7)	4813 (10.1)	4212 (10.3)	2293 (10.5)
Hypertension (%)	32652 (23.5)	7227 (24.8)	11452 (24.1)	9291 (22.8)	4682 (21.5)
Diabetes (%)	5140 (3.7)	1035 (3.6)	1753 (3.7)	1563 (3.8)	789 (3.6)
Cardiovascular disease (%)	6919 (5.0)	1538 (5.3)	2506 (5.3)	1876 (4.6)	999 (4.6)
eGFR (mL/min per 1.73 m2)	93.9 (84.9, 100.9)	93.3 (84.2, 100.7)	93.4 (84.3, 100.4)	94.1 (85.2, 101.0)	95.2 (87.0, 102.2)
Total energy intake (kcal/day)	2015.4 (1689.0, 2388.0)	2118.6 (1788.8, 2499.4)	2027.4 (1701.6, 2390.6)	1966.5 (1646.3, 2331.9)	1946.9 (1629.7, 2320.8)
Vegetables (g/day)	171.2 (88.0, 281.6)	127.5 (66.0, 201.0)	154.4 (76.7, 250.0)	192.5 (99.8, 305.7)	264.8 (158.5, 387.7)
Fruits (g/day)	182.0 (97.5, 294.7)	96.7 (5.0, 182.0)	165.2 (86.7, 271.0)	213.5 (120.0, 325.0)	266.0 (180.0, 380.0)
Unsaturated oils (g/day)	0.0 (0.0, 10.0)	0.0 (0.0, 7.0)	0.0 (0.0, 9.7)	1.8 (0.0, 10.0)	3.3 (0.0, 12.4)
Legumes (g/day)	0.0 (0.0, 17.5)	0.0 (0.0, 0.0)	0.0 (0.0, 8.8)	0.0 (0.0, 23.3)	11.7 (0.0, 67.5)
Nuts (g/day)	0.0 (0.0, 6.7)	0.0 (0.0, 3.0)	0.0 (0.0, 5.2)	0.0 (0.0, 9.0)	3.0 (0.0, 20.0)
Whole grains (g/day)	215.3 (142.2, 302.6)	172.0 (109.3, 240.0)	205.5 (138.0, 291.9)	234.9 (156.0, 320.0)	270.0 (187.0, 353.0)
Fish (g/day)	0.0 (0.0, 50.0)	0.0 (0.0, 0.0)	0.0 (0.0, 40.0)	32.5 (0.0, 70.0)	50.0 (0.0, 100.0)
Beef and lamb (g/day)	0.0 (0.0, 60.0)	40.0 (0.0, 72.0)	0.0 (0.0, 60.0)	0.0 (0.0, 30.0)	0.0 (0.0, 0.0)
Pork (g/day)	7.7 (0.0, 40.2)	35.0 (10.0, 61.8)	15.3 (0.0, 46.0)	0.0 (0.0, 23.0)	0.0 (0.0, 0.0)
Poultry (g/day)	0.0 (0.0, 65.0)	32.5 (0.0, 65.0)	0.0 (0.0, 65.0)	0.0 (0.0, 48.8)	0.0 (0.0, 0.0)
Eggs (g/day)	0.0 (0.0, 30.0)	16.7 (0.0, 50.0)	0.0 (0.0, 33.3)	0.0 (0.0, 18.8)	0.0 (0.0, 0.0)
Dairy (g/day)	250.0 (160.0, 345.0)	260.0 (170.0, 346.7)	260.0 (170.0, 350.0)	248.0 (160.0, 345.4)	225.0 (132.5, 327.5)
Potatoes (g/day)	87.5 (0.0, 175.0)	120.0 (60.0, 180.0)	90.0 (0.0, 175.0)	60.0 (0.0, 133.8)	30.3 (0.0, 90.0)
Added sugar (g/day)	54.8 (36.3, 77.8)	65.7 (47.1, 89.9)	57.5 (39.1, 79.7)	49.9 (33.1, 72.5)	43.0 (27.2, 63.6)

Data were presented as frequency (%), mean ± standard deviation or median (interquartile range).

Abbreviation: BMI, body mass index; eGFR, estimated glomerular filtration rate.

### 3.2 Associations of three PHDIs with incident CKD

**Fig. 2:**
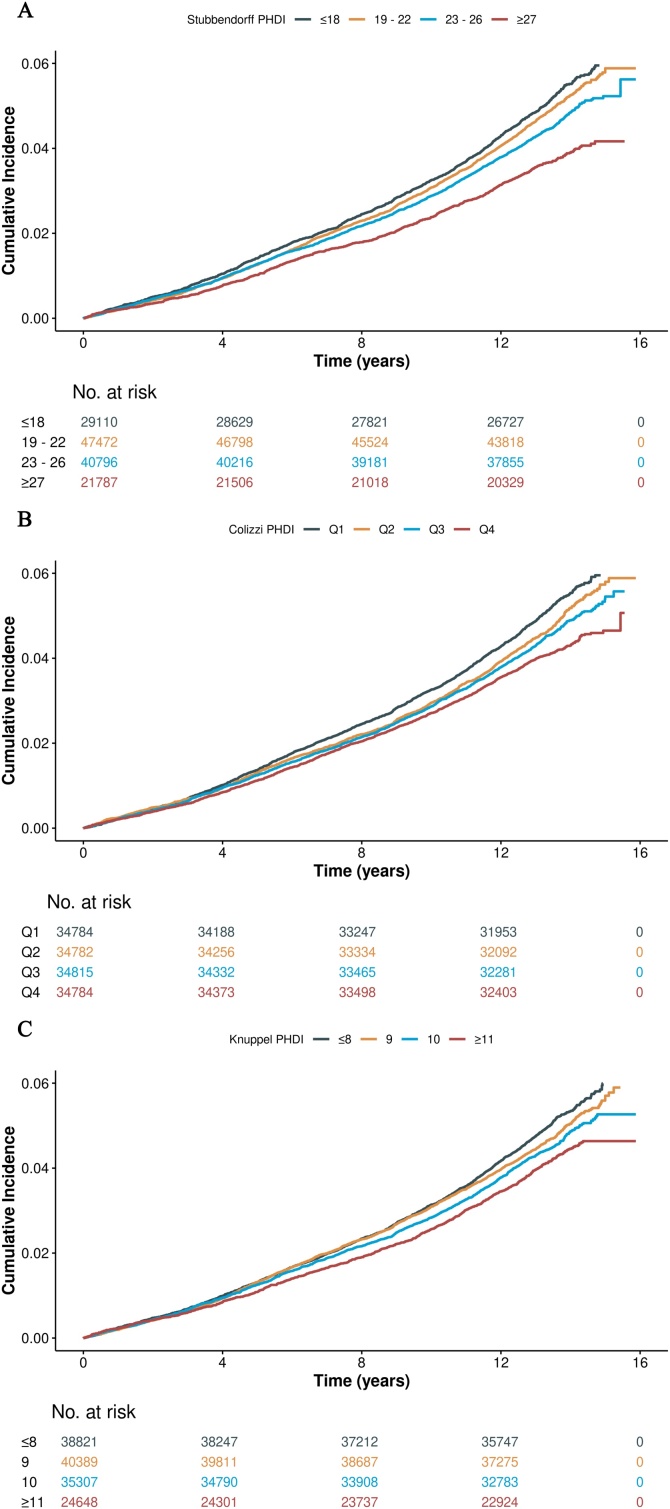
Kaplan–Meier survival curves for incident CKD according to category of Stubbendorff, Colizzi and Knuppel PHDI. The cumulative incidence curves for CKD based on the different PHDIs.(A) Stubbendorff PDHI;(B) Colizzi PHDI;(C) Knuppel PHDI.

During a median follow-up of 13.3 years, 6,391 incident cases of CKD were identified. The adjusted survival curve showed that participants with lower adherence to the planetary health diet exhibited a higher risk of incident CKD across all three different PHDIs (Fig. 2). For the Stubbendorff PHDI, compared with participants in the lowest adherence group (≤18 points), those in the highest adherence group (≥27 points) had an adjusted HR of 0.827 (95% CI, 0.757−0.904) in fully adjusted Cox proportional hazards models. When analyzed as a continuous variable, each additional point in the Stubbendorff PHDI was associated with a 1.3% decreased risk of incident CKD (Table 2). For the Colizzi PHDI, the adjusted HR for the highest adherence group (quartile 4) was 0.865 (95% CI, 0.805−0.929) compared with the lowest adherence group. Each increase in 10-points was associated with a 3.9% lower risk of incident CKD when regarding the Colizzi PHDI as a continuous variable (Table 2). For the Knuppel PHDI, compared with the lowest adherence group (≤8 points), participants in the highest adherence group (≥11 points) had an adjusted HR of 0.891 (95% CI, 0.821−0.996) after full adjustment. Each 1-point increase of Knuppel PHDI was associated with 3.1% decreased risk of incident CKD (Table 2).

**Table 2:** Associations between the different PHDI and risks of CKD.

	Cases/Total	Model 1^a^		Model 2^b^		Model 3^c^	
		HR (95% CI)	*P value*	HR (95% CI)	*P value*	HR (95% CI)	*P value*
**Stubbendorff PHDI**							
≤18	1487/29110	REF		REF		REF	
19–22	2297/47472	0.908 (0.851−0.970)	0.004	0.918 (0.860−0.980)	0.010	0.955 (0.894–1.020)	0.167
23–26	1816/40796	0.837 (0.782−0.897)	<0.001	0.855 (0.798−0.916)	<0.001	0.927 (0.865−0.994)	0.033
≥27	791/21787	0.685 (0.628−0.747)	<0.001	0.710 (0.651−0.775)	<0.001	0.827 (0.757−0.904)	<0.001
*P* for trend		<0.001		<0.001		0.004	
1-point increment in diet index	6391/139165	0.973 (0.968−0.979)	<0.001	0.976 (0.970−0.982)	<0.001	0.987 (0.981−0.993)	<0.001
**Colizzi PHDI**							
Q1	1769/34784	REF		REF		REF	
Q2	1638/34782	0.878 (0.821−0.939)	<0.001	0.902 (0.844−0.965)	0.003	0.924 (0.864−0.989)	0.022
Q3	1569/34815	0.820 (0.765−0.877)	<0.001	0.858 (0.801−0.918)	<0.001	0.906 (0.846−0.970)	0.005
Q4	1415/34784	0.741 (0.691−0.795)	<0.001	0.787 (0.734−0.845)	<0.001	0.865 (0.805−0.929)	<0.001
*P* for trend		<0.001		<0.001		0.004	
10-point increment in diet index	6391/139165	0.921 (0.906−0.938)	<0.001	0.937 (0.921−0.953)	<0.001	0.961 (0.944−0.978)	<0.001
**Knuppel PHDI**							
≤8	1928/38821	REF		REF		REF	
9	1887/40389	0.918 (0.862−0.979)	0.009	0.924 (0.867−0.985)	0.016	0.950 (0.891–1.013)	0.117
10	1574/35307	0.878 (0.821−0.940)	<0.001	0.886 (0.828−0.947)	<0.001	0.931 (0.869−0.997)	0.042
≥11	1002/24648	0.807 (0.747−0.873)	<0.001	0.823 (0.761−0.889)	<0.001	0.891 (0.821−0.966)	0.005
*P* for trend		<0.001		<0.001		0.004	
1-point increment in diet index	6391/139165	0.944 (0.926−0.962)	<0.001	0.949 (0.932−0.967)	<0.001	0.969 (0.950−0.988)	0.002

a Model 1 was adjusted for age, sex, ethnicity;

b Model 2 was adjusted for model 1 plus Townsend deprivation index, education, hypertension, diabetes, cardiovascular disease, cancer;

c Model 3 was adjusted for model 2 plus BMI, eGFR, smoking status, alcohol intake, physical activity, total energy intake.

### 3.3 PHDI, genetic risk, and the risk of CKD

**Fig. 3:**
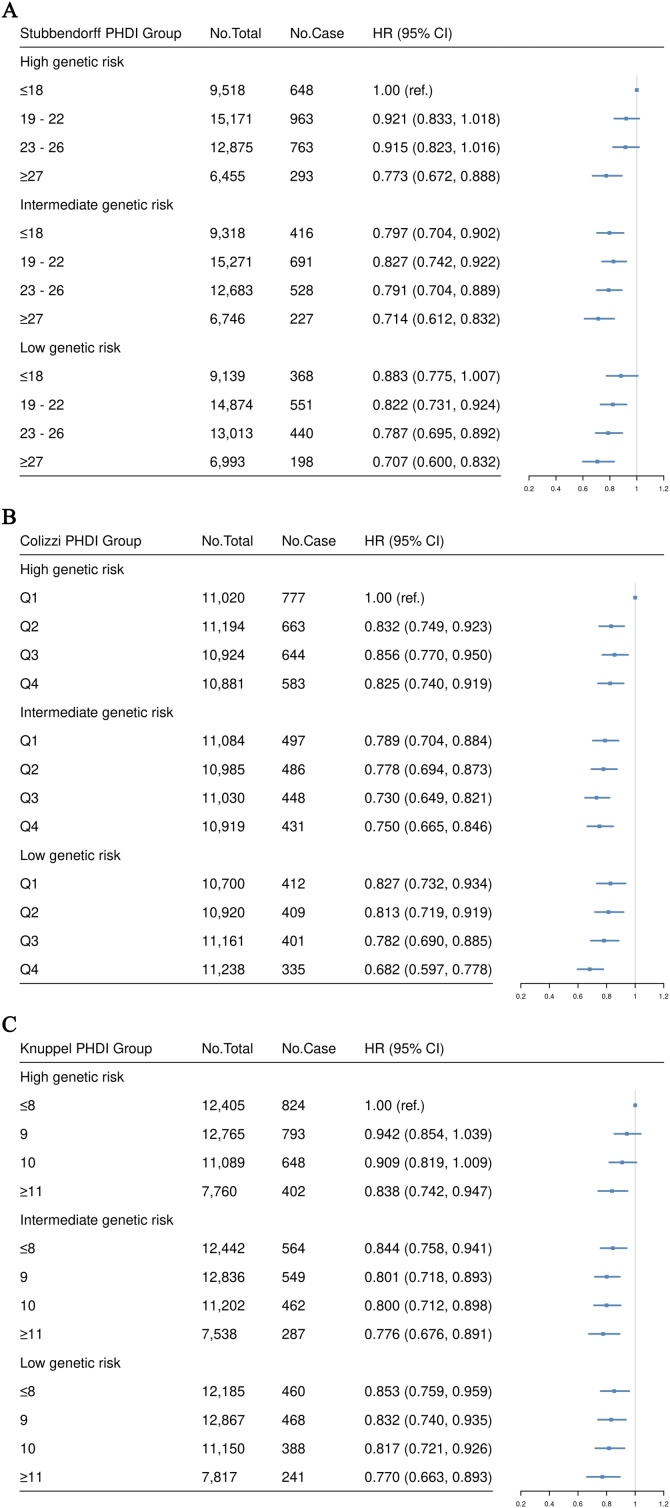
Risk of incident CKD according to different PHDI and genetic risk. (A) Joint effects of Stubbendorff PHDI and genetic risk; (B) Joint effects of Colizzi PHDI and genetic risk; (C) Joint effects of Knuppel PHDI and genetic risk. All adjusted for age, sex, ethnicity, Townsend deprivation index, cardiovascular disease, diabetes, hypertension, BMI, education, smoking status, alcohol intake, physical activity, and total energy intake.

Among the total population, 132,056 participants have the genotype data and the PRS was calculated for each participant. The associations of genetic risk with incident CKD were investigated and the results revealed a positive association between genetic susceptibility and the risk of incident CKD in both categorical and continuous analyses (Table S10). The joint effects of the three different PHDIs and genetic risk on CKD risk were shown in Fig. 3. Participants with highest adherence to the planetary health diet and low genetic risk had the lowest risk of incident CKD. Compared to participants with lowest adherence to planetary health diet and high genetic risk, the HRs (95% CI) of incident CKD for those with highest adherence to planetary health diet and low genetic risk were 0.707 (95% CI, 0.600−0.832), 0.682 (95% CI, 0.597−0.778), and 0.770 (95% CI, 0.663−0.893) for Stubbendorff PHDI, Colizzi PHDI and Knuppel PHDI, respectively.

### 3.4 Several sensitivity and subgroup analyses

When using competing risk models to re-assess the associations between PHDI and incident CKD, similar inverse associations were observed in all the three different PHDIs (Table S11). The associations of PHDI with incident CKD were also consistent after excluding participants diagnosed with CKD within the first three years of follow-up (Table S12), using the follow-up phase which began at the time when the latest dietary questionnaire was completed(Table S13), and excluding participants who completed only one dietary assessment (Table S14). Mediation analysis revealed that BMI (18.5% [95% CI, 12.10 %–31.00 %]) partially mediated the association between Stubbendorff PHDI and incident CKD. The mediating effects of hypertension, cardiovascular disease and diabetes were relatively weak and not significant. Similar results were observed for the Colizzi and Knuppel PHDI (Table S15). In the subgroup analyses, the associations between adherence to planetary health diet and incident CKD were consistent across all the prespecified subgroup factors (Table S16-S18), and no significant interaction was observed between subgroup factors and PHDI for incident CKD. Furthermore, the associations between genetic risk, PHDI and incident CKD were consistent after using the alternative PRS calculation method based on 249 SNPs (Supplementary File 2) reported by Yu et al. (Table S19 and Figure S2). Finally, after imputing missing baseline covariates (Table S20-S22), the results remained consistent across all the three PHDIs (Table S23).

## 4 Discussion

In this study, using data from the large UK Biobank cohort, we investigated the association between three different PHDIs and the risk of CKD. Our findings demonstrated that higher adherence to planetary health diet was associated with lower risk of incident CKD. Furthermore, we examined the combined effects of adherence to planetary health diet and genetic risk, finding that participants with highest adherence to the planetary health diet and low genetic risk had the lowest risk of CKD. These associations were consistent across the three PHDIs and remained robust in several sensitivity analyses.

The planetary health diet, proposed by the EAT-Lancet Commission in 2019, represents a global dietary pattern that balances human health and environmental sustainability [6]. Since its publication, several studies have explored its associations with human health and environmental outcomes. A large European cohort study revealed that shifting from the lower to higher adherence to the planetary health diet could significantly reduce diet-related greenhouse gas emissions up to 50% and prevent premature deaths up to 19 %–63 % [26]. Similarly, a Chinese cohort study reported that each 1 standard deviation increase in PHDI was associated with a 2.2% decrease in greenhouse gas emissions, a 2.3% reduction in land use, and an 8% lower risk of mortality [11]. Additionally, higher adherence to the planetary health diet has been linked to reduced risks of diabetes [12], steatotic liver disease [27], cardiovascular disease [18,28,29], stroke [30], cancer [31], and mental health conditions such as depression, anxiety, and dementia [10,32]. While dietary interventions play a crucial role in the prevention and treatment of CKD [4,33], few studies have examined the associations between the planetary health diet and incident CKD. To our knowledge, this is the first study to evaluate the role of planetary health diet in the development of CKD. Our findings underscore the potential of the planetary health diet in preventing CKD, further enhancing its value for human health.

The translation of the planetary health diet into a dietary score has varied across studies, and there is no consensus on how to quantify adherence to this diet. Our study carefully considered the consistency of scoring methods based on their application in other research cohorts. A previous study systematically compared seven different PHDIs in relation to health and environmental outcomes [18]. Among these, the indexes proposed by Stubbendorff et al. and Colizzi et al. were found to be the most consistent in categorizing participants according to the planetary health diet, as well as their associations with disease, mortality, and greenhouse gas emissions across the validation cohorts. The Stubbendorff PHDI employs an ordinal scale system, while the Colizzi PHDI uses a proportional scoring system. Additionally, the Knuppel PHDI, the first and most widely used index, utilizes a binary system. These three distinct and representative PHDIs were selected to evaluate the robustness of the relationship between adherence to the planetary health diet and the risk of incident CKD in our study. Each scoring methods has unique characteristics. The ordinal scale used by Stubbendorff PHDI make it simple to use and interpret, but may limit the statistical power in discriminating participants. In contrast, the proportional and continuous score used by the Colizzi PHDI retains statistical power but may lack precision due to the semiquantitative nature of the 24-h dietary questionnaire used in the UK Biobank. The Knuppel PHDI, while straightforward to interpret, may lead to unintended consequences due to the establishment of minimum and maximum values, resulting in low consistency across different populations. By combining these approaches, we mitigated the biases associated with their individual limitations, thereby validating and strengthening the robustness of the relationship between adherence to the planetary health diet and the risk of developing CKD, and the differences in predictive performance among the three PHDIs may be attributed to discrepancies in their score construction.

The associations between adherence to planetary health diet and decreased incident CKD risks can be explained by several mechanisms. The planetary health diet primarily recommends a dietary structure rich in plant-based foods, some fish, and limited dairy and meat. Similarly dietary patterns, such as plant-based and vegetarian diets [5], the Mediterranean diet [34] and the DASH diet [35], have also been demonstrated to be associated with lower CKD incidence. First, limiting animal protein intake, particularly from red meat, may reduce the production of the harmful catabolic by-products of protein metabolism, thereby decreasing chronic inflammation, oxidative stress, and endothelial dysfunction [36,37]. Second, reducing excessive carbohydrate intake can lower blood triglyceride levels, increase HDL cholesterol, reduce elevated blood pressure, and improve insulin resistance [38,39]. Third, a plant-based diet enhances alkali intake and reduces endogenous acid production, helping to control metabolic acidosis and prevent declines in kidney function [40]. Furthermore, gut microbiome dysbiosis, a recognized risk factor for CKD development and progression, can be modified by diet [[41], [42], [43]]. Excessive animal protein intake promotes the proliferation of proteolytic bacteria that ferment dietary protein and generate uremic toxins, such as indoxyl sulfate, indole-3 acetic acid, p-cresyl sulfate, and trimethylamine N-oxide. These uremic toxins alter the colonic environment, leading to microbial dysbiosis and disturbances in the intestinal barrier, which interact with the kidneys through inflammatory and fibrotic signaling pathways. In contrast, a plant-dominant, fiber-rich, low-protein diet encourages the growth of saccharolytic bacteria, which compete with proteolytic bacteria, potentially reducing uremic toxin production. Moreover, fiber metabolism boosts the formation of short-chain fatty acids in the gut, which helps maintain intestinal barrier integrity. The increased translocation of short-chain fatty acids into circulation may aid in controlling metabolic acidosis and sustaining metabolic homeostasis [44]. Future research is needed to further compare the planetary health diet with other dietary patterns, elucidating the differences and providing deeper insights into the mechanisms by how the planetary health diet reduces the risk of CKD.

To our knowledge, this is the first study to investigate the interplay between genetic predisposition and adherence to planetary health diet in the development of CKD. No significant interaction was observed between genetic susceptibility and adherence to the planetary health diet in relation to CKD risk. Our findings highlight that the planetary health diet is beneficial for the general population, regardless of genetic predisposition. Furthermore, participants with highest adherence to planetary health diet and low genetic risk has the lowest risk for incident CKD, suggesting that the high genetic risk of CKD can be exacerbated by an unhealthy dietary pattern. These findings underscore the strong potential benefits of adhering to the planetary health diet for reducing the future risk of CKD, irrespective of genetic background.

The strengths of this study include the use of a large prospective cohort with a long-term follow-up and the evaluation of three different PHDIs to assess the associations between adherence to the planetary health diet and CKD risk. The robustness of our findings was further supported by several sensitivity and subgroup analyses. Nevertheless, there are several limitations in the present study. First, as an observational study, our findings may only reveal associations rather than causations. Second, the UK Biobank cohort primarily includes middle-aged, predominantly white participants, limiting the generalizability of our findings to other age groups or ethnicities. Third, only 24 -h dietary recall surveys were conducted in UK Biobank, rather than the more accurate 7-day dietary surveys. Although previous studies have validated that the Oxford WebQ questionnaire, which used by UK Biobank, exhibits good correlation with traditional dietary evaluation methods, and repeated assessments (2–4 times) have been shown to further enhance measurement accuracy and reduce random variability [45], 37.1% of participants included in our study completed only one 24-h dietary assessment, which may not enable us to fully capture the changes in dietary patterns and habitual intake. To address these concerns, we conducted a sensitivity analysis excluding participants with only one dietary assessment, and the results remained consistent. Fourth, the changes of eGFR and urine albumin-creatine ratio during the follow-up could not be obtained for most participants in the UK biobank, the diagnosis of incident CKD was primarily based on claims codes and self-reported data. Furthermore, albuminuria concentration cannot fully substitute for the urine albumin-creatinine ratio, nor is it the gold standard for CKD diagnosis recommended by the KDIGO guidelines [46], these may potentially introduce misclassification bias of CKD diangnosis and skew the results. Fifth, participants with missing baseline data were excluded from the primarily analysis, potentially leading to selection bias. However, sensitivity analyses after imputing missing data yielded consistent results, indicating the reliability of our primary findings. Finally, none of the current scoring methods for the planetary health diet perfectly align with all the food items in the UK Biobank's 24 -h dietary questionnaire. Therefore, further research is needed to more comprehensively utilize dietary assessment information to fully elucidate the complex relationship between the planetary health diet and CKD risk.

## 5 Conclusions

In conclusion, based on the large, population-based UK biobank cohort, we demonstrated that higher adherence to the planetary health diet was associated with lower risk of CKD, with these effects enhanced by genetic susceptibility. These results suggest that promoting this healthy and sustainable dietary pattern could play a key role in preventing CKD.

## CRediT authorship contribution statement

**Duo Lv**: Methodology, Software, Formal analysis, Visualization, Writing-original draft, Data curation, Writing-review & editing. **Tingting Wang**: Methodology, Software, Formal analysis, Visualization, Writing-review & editing. **Jiayao Fan**: Methodology, Software, Formal analysis, Writing-review & editing. **Dongsheng Hong:** Methodology, Software, Writing-review & editing. **Zhiyi Chen**: Formal analysis, Visualization, Writing-original draft, Writing-review & editing. **Qianchun Xu**: Formal analysis, Visualization, Writing-original draft, Writing-review & editing**Dan Zhou**: Conceptualization, Project administration, Supervision, Writing-review & editing. **Xishao Xie**: Conceptualization, Funding acquisition, Project administration, Supervision, Writing-review & editing.

## Ethics approval

The UK Biobank study was approved by the North West Multi-center Research Ethics Committee (21/NW/0157). This study was conducted under UK Biobank application numbers 102158.

## Declaration of Generative AI and AI-assisted technologies in the writing process

Not applicable.

## Funding sources

This work was supported by grants from the National Natural Science Foundation of China (No. 82200842) and the Zhejiang Provincial Natural Science Foundation of China (No. LZ22H050001).

## Data availability

No datasets were generated or analysed during the current study. The data used in this study were obtained from the UK Biobank resource. Researchers can access the data used in this study through the UK Biobank Consortium website.

## Declaration of competing interest

The authors declare that they have no known competing financial interests or personal relationships that could have appeared to influence the work reported in this paper.
