# Supplementary material for: Planetary health diet index, genetic susceptibility and incident chronic kidney disease: a cohort study from the UK Biobank
Source: J Nutr Health Aging. 2026 Jan 14;30(3):100777. doi: 10.1016/j.jnha.2026.100777 (PMC12835594; doi:10.1016/j.jnha.2026.100777)
Supplement: Supplementary file 1 [file mmc1.docx]

**Supplementary materials**

**Methods S1.** Penalized splines

**Methods S2.** Imputation method

**Figure S1.** Associations of Stubbendorff, Colizzi and Knuppel PHDI with risks of incident CKD in Cox models with penalized splines.

**Figure S2.** Risk of incident CKD according to different PHDI and genetic risk (based on the SNPs from Yu et al.).

**Table S1.** Definition of food items and field ID used in this study.

**Table S2.** Development of the Stubbendorff PHDI based on the criterion from Stubbendorff et al.

**Table S3.** Development of the Colizzi PHDI for men based on the criterion from Colizzi et al.

**Table S4.** Development of the Colizzi PHDI for women based on the criterion from Colizzi et al.

**Table S5.** Development of the Knuppel PHDI based on the criterion from Knuppel et al.

**Table S6.** Definitions and codes for chronic kidney disease in the UK Biobank.

**Table S7.** Definitions and codes for cardiovascular disease, hypertension, diabetes, and cancer in the UK Biobank.

**Table S8.** Baseline characteristics of the study participants according to categories of the Colizzi PHDI.

**Table S9.** Baseline characteristics of the study participants according to categories of the Knuppel PHDI.

**Table S10.** Risk of incident CKD according to different genetic risk based on SNPs from Wuttke et al.

**Table S11.** Associations between the different PHDI and risks of CKD using competing risk models.

**Table S12.** Associations between the different PHDI and risks of CKD after excluding individuals diagnosed with CKD within the first three years of follow-up.

**Table S13.** Associations between the different PHDI and risks of CKD using follow-up phases which began at the time of the completion of the latest dietary questionnaire.

**Table S14.** Associations between the different PHDI and risks of CKD excluding participants who completed only one the 24-hour dietary questionnaire

**Table S15.** Mediating effects of BMI, CVD, diabetes and hypertension in the associations between the different PHDIs and risks of CKD.

**Table S16.** Subgroup analysis of associations between the Stubbendorff PHDI and risks of CKD.

**Table S17.** Subgroup analysis of associations between the Colizzi PHDI and risks of CKD.

**Table S18.** Subgroup analysis of associations between the Knuppel PHDI and risks of CKD.

**Table S19.** Risk of incident CKD according to different genetic risk base on the SNPs from Yu et al.

**Table S20.** Baseline characteristics of the study participants according to categories of the Stubbendorff PHDI after imputation.

**Table S21.** Baseline characteristics of the study participants according to categories of the Colizzi PHDI after imputation.

**Table S22.** Baseline characteristics of the study participants according to categories of the Knuppel PHDI after imputation.

**Table S23.** Associations between the different PHDI and risks of CKD after imputation.

**Methods S1. Penalized splines**

We fit Cox models with penalized splines to explore the nonlinear associations of these three different planetary health diet indexes with incident CKD. In particular, nonparametric smoothers were used for estimating the nonlinear relationship between a continuous predictor (specifically, our planetary health diet index) and hazard ratio. The hazard function for the i-th participant in the j-th group was calculated as^1,2^:

$$\lambda_{j, i}(t)=\lambda_{j, 0}(t)exp(Z_{i}(t))\beta_{Z}+s(X_{i}(t), \mathrm{df}_{i}))$$

where λ*_j.0_*(t) denotes an unspecified underlying hazard function; *β_z_* is a vector of parameters for the covariates Z_i_(t), and s(X_i_(t), df_i_) is a df_i_-degree penalized spline model for the covariate of our main interest X_i_(t).

We set the degree of freedom as 3 when fitting the three different PHDIs, based on the Akaike information criterion (AIC). The associations of planetary health diet index with incident CKD were analyzed in regression models adjusted for age, sex, ethnicity, Townsend scores, educational level, baseline comorbidities (including hypertension, diabetes, cardiovascular disease, and cancer), BMI, eGFR, smoking status, alcohol intake, physical activity and total energy intake.

**Methods S2. Imputation method**

Percentages of missing data per variable are identified; specifically, 0.4% were missing ethnicity, 8.6% missing education level, 0.1% missing Townsend index, 0.3% missing BMI, 0.1% missing smoking status, 0.1% missing alcohol intake, 17.3% missing physical activity and 5.7% missing eGFR. The missing data were imputed using the multiple imputation with chained equation, and 10 imputed datasets was created for the cohort. Effect estimates were computed separately and then combined according to Rubin’s rules^3^.

**Figure S1. Associations of** **Stubbendorff, Colizzi and Knuppel PHDI with risks of incident CKD in Cox models with penalized splines.**


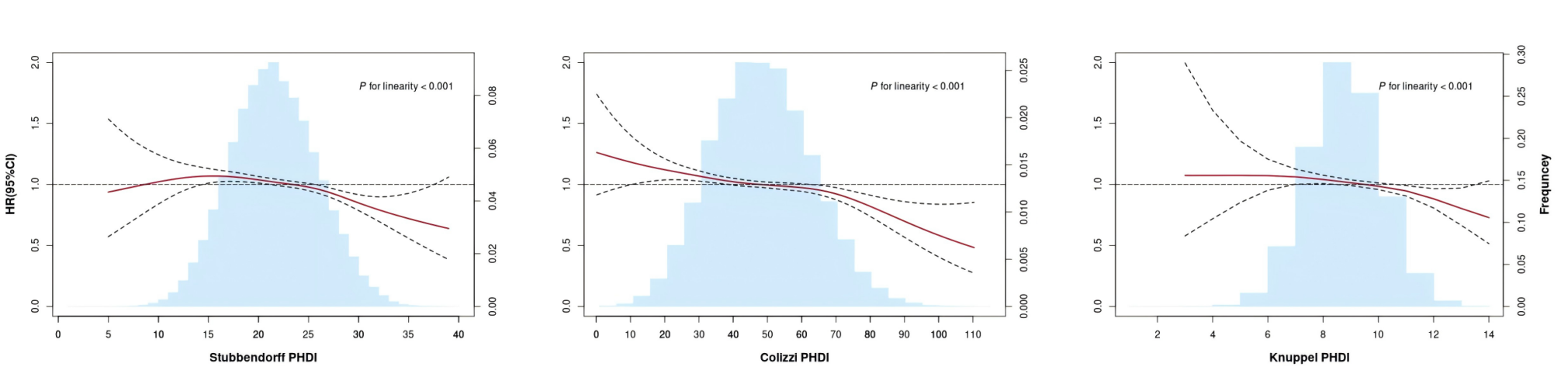


Cox models with penalized splines were adjusted for age, sex, ethnicity, Townsend deprivation index, cardiovascular disease, diabetes, hypertension, BMI, education, smoking status, alcohol intake, physical activity, and total energy intake.

**Figure S2. Risk of incident CKD according to different PHDI and genetic risk.**


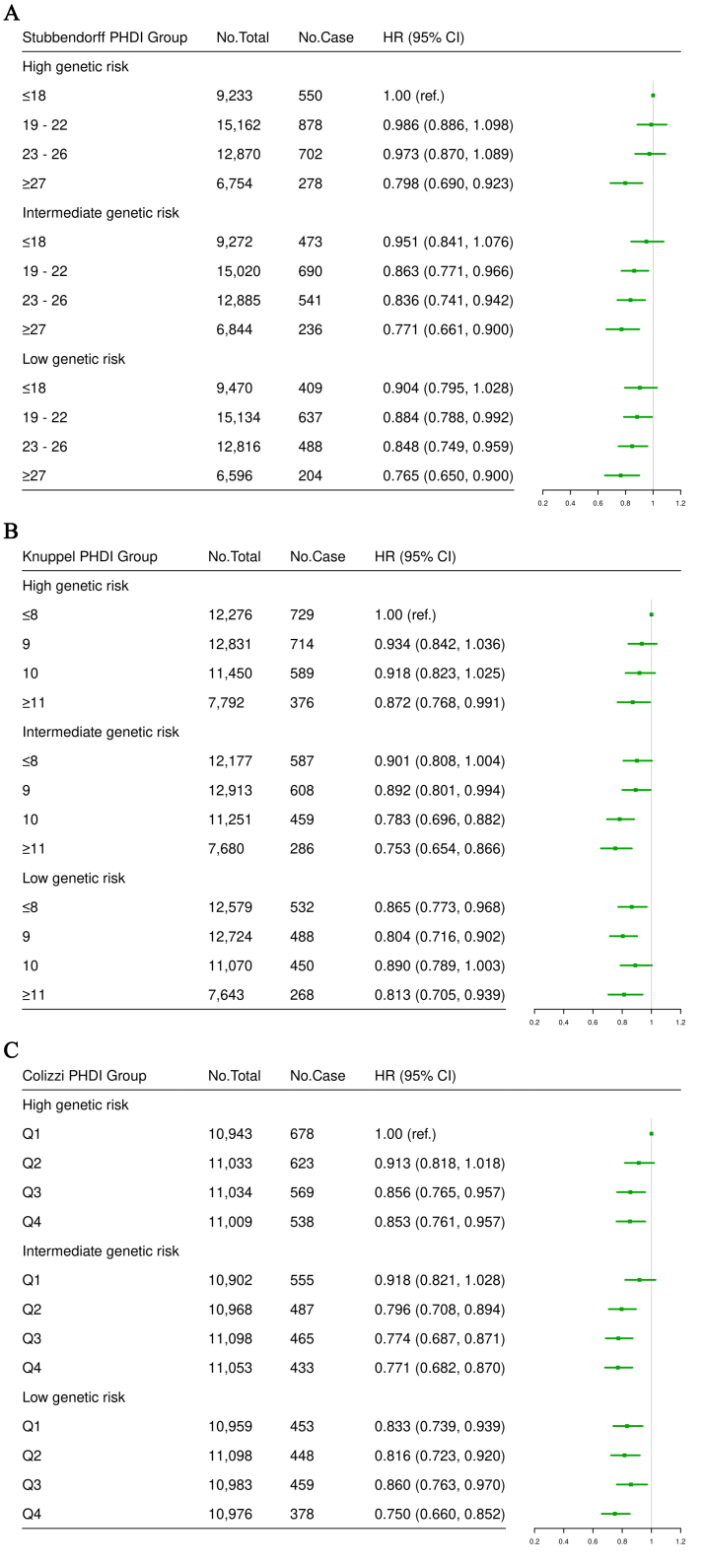


(A) Joint effects of Stubbendorff PHDI and genetic risk; (B) Joint effects of Colizzi PHDI and genetic risk; (C) Joint effects of Knuppel PHDI and genetic risk. All adjusted for age, sex, ethnicity, Townsend deprivation index, cardiovascular disease, diabetes, hypertension, BMI, education, smoking status, alcohol intake, physical activity, and total energy intake.

**Table S1. Definition of food items and field ID used in this study.**

| **Component** | **Food items (Field ID)^b^** |
| --- | --- |
| **Whole** **grains^a^** |  |
| Rice, wheat, corn, and other | Biscuit cereal (26075); Biscuits (26068); Bran cereal (26076); Mixed bread (50/50), brown and seeded (26071); Savoury crackers (26083); White bread (26073); White pasta and rice (26113); Whole meal bread (26074); Other cereal (sugar) (26079); Whole meal pasta, brown rice and other whole grains (26114); Muesli (26105); Other bread (26072); Oat cereal (non sugar) (26077); Oat cereal (sugar) (26078); Porridge (100770)^c^; Pizza (26116) |
| **Tubers** **and** **starchy** **vegetables** |  |
| Potatoes | Fried/roast potatoes (26119); Mashed potatoes (26120); Potatoes and sweet potatoes (baked/boiled) (26118) |
| **Vegetables** |  |
| All vegetables | Other vegetables, including mushrooms, fruiting and mixed vegetables (26146); Green leafy/cabbages (26098); Allium vegetables (26065); Peas and sweetcorn (26115); Raw salad (26123); Root vegetables (26125); Tomatoes (26143); VegTable Sside dishes (26147); Cucumber (104210)^c^ |
| **Fruits** |  |
| All fruits | Plum (104580)^c^; Prune (104420)^c^; Berries (26090); Citrus (26091); Other fruit (26093); Apples and pears (26089) |
| **Dairy** **foods** |  |
| Whole milk or derivative equivalents | Semi skimmed milk (26131); Skimmed milk and cholesterol-lowering milk (26133); Whole milk (26150); Full fat yogurt (26096); High fat cheese (26099); Low fat yogurt (26102); Medium and low fat cheese (26103); Rice/oat milk (26124) |
| **Protein** **sources** |  |
| Beef, lamb, pork | Beef (26066); Pork (26117); Lamb (26100); Other meat, offal (26104); Processed meat (26122) |
| Chicken, other poultry | Poultry (26121); Breaded/battered chicken (26069) |
| Eggs | Egg and egg dishes (26088) |
| Fish | Breaded/battered fish (26070); Oily fish (26109); White fish and tinned tuna (26149); Shellfish (26132) |
| *Legumes* |  |
| Dry beans, lentils, peas | Legumes and pulses (26101) |
| Soy foods | Meat substitutes - soy (26137); Meat substitutes-vegetarian (26145) |
| Peanuts or tree nuts | Unsalted nuts and seeds (26107); Salted nuts and seeds (26108) |
| **Added** **fats** |  |
| Palm oil, unsaturated oils, dairy fats (incl.in milk), lard or tallow | Monounsaturated fatty acids (26032); n-3 fatty acids (26015); n-6 fatty acids (26016); Saturated fatty acids (26104); Olive oil (drizzling/dunking) (20110); Plant-based spread lower fat (26111); Plant-based spread normal (26112); Cream (26154); Animal fat spread lower fat (26062); Animal fat spread normal (26063) |
| **Added** **sugars** |  |
| Added sugar | Free sugar (26012) |

^a^Reference diet refers to dry, raw weight.

^b^The food items (Field ID) based on food group classification was described by Piernas et al.^4^

^c^The number of predefined portion size was defined using the UK’s standard food composition database, the concrete weight was calculated by multiplying the portion size by the number of portions consumed for each food item^5^.

**Table S2. Development of the Stubbendorff PHDI based on the criterion from Stubbendorff et al.^6^**

| **Food components** | **Recommend intake^b^**  **(reference interval)** | **3 points** | **2 points** | **1 point** | **0 point** |
| --- | --- | --- | --- | --- | --- |
|  |  |  |  |  |  |
| Vegetables | 300 (200-600) | >300 | 200-300 | 100-200 | <100 |
| Fruits | 200 (100-300) | >200 | 100-200 | 50-100 | <50 |
| Unsaturated oils | 40 (20-80) | >40 | 20-40 | 10-20 | <10 |
| Legumes | 75 (0-150) | >75 | 37.5-75 | 18.75-37.5 | <18.75 |
| Nuts | 50 (0-100) | >50 | 25-50 | 12.5-25 | <12.5 |
| Whole grains^a^ | 232 | >232 | 116-232 | 58-116 | <58 |
| Fish | 28 (0-100) | >28 | 14-28 | 7-14 | <7 |
| Beef and lamb | 7 (0-14) | <7 | 7-14 | 14-28 | >28 |
| Pork | 7 (0-14) | <7 | 7-14 | 14-28 | >28 |
| Poultry | 29 (0-58) | <29 | 29-58 | 58-116 | >116 |
| Eggs | 13 (0-25) | <13 | 13-25 | 25-50 | >50 |
| Dairy | 250 (0-500) | <250 | 250-500 | 500-1000 | >1000 |
| Potatoes | 50 (0-100) | <50 | 50-100 | 100-200 | >200 |
| Added sugars | 31 (0-31) | <31 | 31-62 | 62-124 | >124 |

^a^Reference diet refers to dry, raw weight.

^b^Recommend intakes from the planetary health diet were according to Willett et al.^7^ and based on an energy intake of 2500 kcal/day.

**Table S3. Development of the Colizzi PHDI for men based on the criterion from Colizzi et al.^8^**

| **Food Group** | **Recommend intake^b^**  **(g/day)** | **Minimum score (0 points)** | **Proportional score** | **Maximum points  (10 points)** | **Proportional score** |
| --- | --- | --- | --- | --- | --- |
| **Whole Grains^a^** |  |  |  |  |  |
| Rice, wheat, corn, and other | 232 (or 60% of total energy) | 0 g/d | 0-232 g/d | ≥232 g/d |  |
| **Vegetables** |  |  |  |  |  |
| All vegetables | 300 | 0 g/d | 0-300 g/d | ≥300 g/d |  |
| **Fruits** |  |  |  |  |  |
| All fruit | 200 | 0 g/d | 0-200 g/d | ≥200 g/d |  |
| **Tubers or starchy vegetables** |  |  |  |  |  |
| Potatoes and cassava | 50 | 0 g/d | 0-50 g/d | 50-100 g/d | 100-150 g/d |
| **Dairy foods** |  |  |  |  |  |
| Whole milk or derivative equivalents | 250 | 0 g/d | 0-250 g/d | 250-500 g/d | 500-750 g/d |
| **Protein Sources** |  |  |  |  |  |
| Dry beans, lentils, and peas | 50 | 0 g/d | 0-50 g/d | ≥50 g/d |  |
| Soy foods | 25 | 0 g/d | 0-25 g/d | ≥25 g/d |  |
| Beef, lamb and pork | 14 | ≥14 g/d | 14 -0 g/d | 0 g/d |  |
| Chicken and other poultry | 29 | 0 g/d | 0-29 g/d | 29-58 g/d | 58-88 g/d |
| Eggs | 13 | 0 g/d | 0-13 g/d | 13-25 g/d | 25-38 g/d |
| Fish | 28 | 0 g/d | 0-28 g/d | 28-100 g/d | 100-128 g/d |
| Nuts | 50 | 0 g/d | 0-50 g/d | 50-100 g/d | 100-150 g/d |
| **Added sugars** | 31 | ≥31 g/d | 31-0 g/d | 0 g/d |  |
| **Added fats** |  |  |  |  |  |
| Palm oil, unsaturated oils, dairy fats (incl. in milk), lard or tallow | Individual limits for  palm oil, unsaturated fat, dairy fats, lard and tallow | No consumption of unsaturated fat OR ratio of unsaturated fat to saturated fat of ≤0.6 | | No consumption of unsaturated fat OR ratio of unsaturated fat to saturated fat of ≥13 | |

^a^Reference diet refers to dry, raw weight.

^b^Recommend intakes from the planetary health diet were according to Willett et al.^7^ and based on an energy intake of 2500 kcal/day for men.

**Table S4. Development of the Colizzi PHDI for women based on the criterion from Colizzi et al.^8^**

| **Food Group** | **Recommend intake  (g/day)** | **Minimum score  (0 points)** | **Proportional score** | **Maximum points  (10 points)** | **Proportional score** |
| --- | --- | --- | --- | --- | --- |
| **Whole Grains*** |  |  |  |  |  |
| Rice, wheat, corn, and other | 186 (or 60% of total energy) | 0 g/d | 0-186 g/d | ≥186 g/d |  |
| **Vegetables** |  |  |  |  |  |
| All vegetables | 240 | 0 g/d | 0-240 g/d | ≥240 g/d |  |
| **Fruits** |  |  |  |  |  |
| All fruit | 160 | 0 g/d | 0-160 g/d | ≥160 g/d |  |
| **Tubers or starchy vegetables** |  |  |  |  |  |
| Potatoes and cassava | 40 | 0 g/d | 0-40 g/d | 40-80 g/d | 80-120 g/d |
| **Dairy foods** |  |  |  |  |  |
| Whole milk or derivative equivalents | 200 | 0 g/d | 0-200 g/d | 200-400 g/d | 400-600 g/d |
| **Protein Sources** |  |  |  |  |  |
| Dry beans, lentils, and peas | 40 | 0 g/d | 0-40 g/d | ≥40 g/d |  |
| Soy foods | 20 | 0 g/d | 0-20 g/d | ≥20 g/d |  |
| Beef, lamb and pork | 12 | ≥12 g/d | 12 -0 g/d | 0 g/d |  |
| Chicken and other poultry | 23 | 0 g/d | 0-23 g/d | 23-46 g/d | 46-69 g/d |
| Eggs | 10 | 0 g/d | 0-10 g/d | 10-20 g/d | 20-30 g/d |
| Fish | 22 | 0 g/d | 0-22 g/d | 22-80 g/d | 80-102 g/d |
| Nuts | 40 | 0 g/d | 0-40 g/d | 40-80 g/d | 80-120 g/d |
| **Added sugars** | 25 | ≥25 g/d | 25-0 g/d | 0 g/d |  |
| **Added fats** |  |  |  |  |  |
| Palm oil, unsaturated oils, dairy fats (incl. in milk), lard or tallow | Individual limits for palm oil, unsaturated fat,  dairy fats, lard and tallow | No consumption of unsaturated fat OR ratio of unsaturated fat to saturated fat of ≤0.5 | | No consumption of unsaturated fat OR ratio of unsaturated fat to saturated fat of ≥13 | |

^a^Reference diet refers to dry, raw weight.

^b^Recommend intakes from the planetary health diet were according to Willett et al.^7^ and based on an energy intake of 2000 kcal/day for women.

**Table S5. Development of the Knuppel PHDI based on the criterion from Knuppel et al.^9^**

| **Component** | **Criteria for minimum**  **score of 0^b^** | **Criteria for maximum**  **score of 1** |
| --- | --- | --- |
| **Whole grains^a^** |  |  |
| Rice, wheat, corn, and other | >232 g/day | ≤232 g/day |
| **Tubers and starchy vegetables** |  |  |
| Potatoes | >100g/day | ≤100 g/day |
| **Vegetables** |  |  |
| All vegetables | <200g/day | ≥200g/day |
| **Fruits** |  |  |
| All fruits | <100g/day | ≥100g/day |
| **Dairy foods** |  |  |
| Whole milk or derivative equivalents | >500g/day | ≤500g/day |
| **Protein sources** |  |  |
| Beef, lamb, pork | >28g/day | ≤28g/day |
| Chicken, other poultry | >58g/day | ≤58g/day |
| Eggs | >25g/day | ≤25g/day |
| Fish | >100g/day | ≤100g/day |
| **Legumes** |  |  |
| Dry beans, lentils, peas | >100g/day | ≤100 g/day |
| Soy foods | >50g/day | ≤50g/day |
| Peanuts or tree nuts | <25g/day | ≥25g/day |
| **Added fats** |  |  |
| Palm oil, unsaturated oils, dairy fats (incl.in milk), lard or tallow |  | Ratio of 0.8 for unsaturated: saturated fat intake |
| **Added sugars** | >31g/day | ≤31g/day |

^a^Reference diet refers to dry, raw weight.

^b^Recommend intakes from the planetary health diet were according to Willett et al.^7^

**Table S6. Definitions and codes for chronic kidney disease in the UK Biobank.**

| **Disease** | **ICD-10** | **ICD-9** | **OPCS-4** |
| --- | --- | --- | --- |
| Chronic kidney disease | D59.3, E10.2, E11.2, E12.2, E13.2, E14.2, E85.3, I12, I13, I15.0, M10.3, N02, N03, N04, N05, N06, N07, N08, N11, N12, N13, N14, N15, N16, N18, N19, N25, N26, N28.0, N28.8, N28.9, N39.1, O10.2, O11, O12.1, O12.2, O14, Q60, R39.2, T86.1, Z49.0, Z94.0 | 403, 581, 582, 583, 585, 586, 587, 588, 589, 591, 642, 2503, 7530, 7531, 7532, 7533, 7910 | L74.1-74.6, L74.8-74.9, M01.2-01.9, M02.3, M08.4, M17.2, M17.4, M17.8-17.9, X40.2, X40.5-40.6, X41.1-41.2 |

Abbreviations: ICD-10, International Classification of Diseases-10th Revision; ICD-9, International Classification of Diseases-9th Revision; OPCS-4, Office of Population Censuses and Surveys Classification of Interventions and Procedures.

**Table S7. Definitions and codes for cardiovascular disease, hypertension, diabetes, and cancer in the UK Biobank.**

| **Comorbidities** | **ICD-10** | **ICD-9** | **OPCS-4** |
| --- | --- | --- | --- |
| Cardiovascular disease | I00-I99, Z95.1, Z95.5 | 390-459 | K40-K46, K49, K50, K75,A05.2-A05.4,  L34.3, L35.1, L35.3, K62.1-K62.3 |
| Hypertension | ICD-10: I11-I13, I15, O10 | 401-405 |  |
| Diabetes | E10-E14 | 250 |  |
| Cancer | C00-C97, Z85 | 140-209, V10 |  |

Abbreviations: ICD-10, International Classification of Diseases-10th Revision; ICD-9, International Classification of Diseases-9th Revision; OPCS-4, Office of Population Censuses and Surveys Classification of Interventions and Procedures.

**Table S8. Baseline characteristics of the study participants according to categories of the Colizzi PHDI.**

| **Characteristics** | **Total** | **Categories of the Colizzi PHDI** | | | |
| --- | --- | --- | --- | --- | --- |
|  |  | **Q1** | **Q2** | **Q3** | **Q4** |
| No. participants | 139165 | 34784 | 34782 | 34815 | 34784 |
| Age (years) | 56.0 (49.0, 62.0) | 55.0 (47.0, 61.0) | 56.0 (49.0, 62.0) | 57.0 (49.0, 62.0) | 57.0 (50.0, 62.0) |
| Sex (male, %) | 65058 (46.7) | 18988 (54.6) | 16536 (47.5) | 15465 (44.4) | 14069 (40.4) |
| Ethnicity (%) |  |  |  |  |  |
| White | 133677 (96.1) | 33222 (95.5) | 33521 (96.4) | 33521 (96.3) | 33413 (96.1) |
| Asian | 1489 (1.1) | 583 (1.7) | 358 (1.0) | 314 (0.9) | 234 (0.7) |
| Black | 2195 (1.6) | 497 (1.4) | 471 (1.4) | 556 (1.6) | 671 (1.9) |
| Other | 1804 (1.3) | 482 (1.4) | 432 (1.2) | 424 (1.2) | 466 (1.3) |
| Education (%) |  |  |  |  |  |
| College or University degree | 68168 (49.0) | 14250 (41.0) | 16349 (47.0) | 17814 (51.2) | 19755 (56.8) |
| Other | 70997 (51.0) | 20534 (59.0) | 18433 (53.0) | 17001 (48.8) | 15029 (43.2) |
| Townsend deprivation index | -2.4 (-3.8, -0.1) | -2.4 (-3.7, 0.0) | -2.4 (-3.8, -0.2) | -2.4 (-3.8, -0.2) | -2.4 (-3.8, 0.0) |
| BMI (%, kg/m2) |  |  |  |  |  |
| Mean (SD) | 26.6 (4.4) | 27.3 (4.6) | 26.8 (4.5) | 26.5 (4.3) | 26.0 (4.2) |
| <18.5 | 730 (0.5) | 131 (0.4) | 164 (0.5) | 173 (0.5) | 262 (0.8) |
| 18.5-24.9 | 54203 (38.9) | 11349 (32.6) | 12897 (37.1) | 14162 (40.7) | 15795 (45.4) |
| 25-29.9 | 58069 (41.7) | 15247 (43.8) | 14833 (42.6) | 14456 (41.5) | 13533 (38.9) |
| ≥30 | 26163 (18.8) | 8057 (23.2) | 6888 (19.8) | 6024 (17.3) | 5194 (14.9) |
| Smoking (n, %) |  |  |  |  |  |
| Never | 80439 (57.8) | 19190 (55.2) | 20227 (58.2) | 20379 (58.5) | 20643 (59.3) |
| Previous | 48296 (34.7) | 11832 (34.0) | 11943 (34.3) | 12203 (35.1) | 12318 (35.4) |
| Current | 10430 (7.5) | 3762 (10.8) | 2612 (7.5) | 2233 (6.4) | 1823 (5.2) |
| Alcohol intake (%) |  |  |  |  |  |
| Never | 7644 (5.5) | 1923 (5.5) | 1842 (5.3) | 1885 (5.4) | 1994 (5.7) |
| Special occasions only | 11979 (8.6) | 3030 (8.7) | 3048 (8.8) | 2937 (8.4) | 2964 (8.5) |
| 1-3 times/month | 14771 (10.6) | 3728 (10.7) | 3680 (10.6) | 3618 (10.4) | 3745 (10.8) |
| 1-2 times/week | 34294 (24.6) | 8413 (24.2) | 8680 (25.0) | 8633 (24.8) | 8568 (24.6) |
| 3-4 times/week | 36856 (26.5) | 8804 (25.3) | 9031 (26.0) | 9401 (27.0) | 9620 (27.7) |
| Daily or almost daily | 33621 (24.2) | 8886 (25.5) | 8501 (24.4) | 8341 (24.0) | 7893 (22.7) |
| Physical activity (%) |  |  |  |  |  |
| Low | 24808 (17.8) | 7397 (21.3) | 6455 (18.6) | 5785 (16.6) | 5171 (14.9) |
| Moderate | 59479 (42.7) | 14672 (42.2) | 14885 (42.8) | 14969 (43.0) | 14953 (43.0) |
| High | 54878 (39.4) | 12715 (36.6) | 13442 (38.6) | 14061 (40.4) | 14660 (42.1) |
| Cancer (%) | 14135 (10.2) | 3375 (9.7) | 3531 (10.2) | 3570 (10.3) | 3659 (10.5) |
| Hypertension (%) | 32652 (23.5) | 8607 (24.7) | 8303 (23.9) | 8053 (23.1) | 7689 (22.1) |
| Diabetes (%) | 5140 (3.7) | 1400 (4.0) | 1337 (3.8) | 1248 (3.6) | 1155 (3.3) |
| Cardiovascular disease (%) | 6919 (5.0) | 1889 (5.4) | 1747 (5.0) | 1713 (4.9) | 1570 (4.5) |
| eGFR (mL/min per 1.73 m^2^) | 93.9 (84.9, 100.9) | 93.9 (84.7, 101.3) | 93.5 (84.4, 100.7) | 93.8 (84.9, 100.6) | 94.3 (85.7, 101.1) |
| Total energy intake (kcal/day) | 2015.4 (1689.0, 2388.0) | 1965.9 (1602.4, 2377.8) | 2012.4 (1675.8, 2387.8) | 2021.3 (1709.4, 2382.8) | 2053.1 (1756.1, 2400.7) |
| Whole grains (g/day) | 215.3 (142.2, 302.6) | 166.0 (102.0, 273.0) | 207.0 (136.3, 301.5) | 226.1 (158.0, 307.0) | 246.3 (182.2, 321.9) |
| Potatoes (g/day) | 87.5 (0.0, 175.0) | 90.0 (0.0, 180.0) | 90.0 (0.0, 175.0) | 87.5 (11.2, 145.0) | 83.0 (43.8, 116.7) |
| Vegetables (g/day) | 171.2 (88.0, 281.6) | 92.5 (0.0, 187.1) | 156.0 (80.5, 263.0) | 191.5 (112.4, 297.8) | 236.0 (153.8, 338.0) |
| Fruits (g/day) | 182.0 (97.5, 294.7) | 100.0 (0.0, 200.0) | 172.0 (96.7, 282.0) | 210.0 (117.5, 314.0) | 237.5 (158.4, 345.0) |
| Dairy (g/day) | 250.0 (160.0, 345.0) | 210.0 (115.0, 330.0) | 250.0 (162.5, 347.5) | 261.7 (177.3, 348.3) | 269.4 (191.7, 348.0) |
| Red meat (g/day) | 46.0 (0.0, 92.0) | 76.0 (23.0, 120.0) | 50.0 (0.0, 97.8) | 40.0 (0.0, 80.0) | 23.0 (0.0, 60.0) |
| Poultry (g/day) | 0.0 (0.0, 65.0) | 0.0 (0.0, 65.0) | 0.0 (0.0, 65.0) | 0.0 (0.0, 65.0) | 32.5 (0.0, 50.0) |
| Eggs (g/day) | 0.0 (0.0, 30.0) | 0.0 (0.0, 25.0) | 0.0 (0.0, 31.2) | 0.0 (0.0, 33.3) | 12.5 (0.0, 30.0) |
| Fish (g/day) | 0.0 (0.0, 50.0) | 0.0 (0.0, 0.0) | 0.0 (0.0, 50.0) | 25.0 (0.0, 60.0) | 35.0 (7.5, 60.0) |
| Legumes | 0.0 (0.0, 11.7) | 0.0 (0.0, 0.0) | 0.0 (0.0, 0.0) | 0.0 (0.0, 17.5) | 8.8 (0.0, 35.0) |
| Soy food (g/day) | 0.0 (0.0, 0.0) | 0.0 (0.0, 0.0) | 0.0 (0.0, 0.0) | 0.0 (0.0, 0.0) | 0.0 (0.0, 0.0) |
| Nuts (g/day) | 0.0 (0.0, 6.7) | 0.0 (0.0, 0.0) | 0.0 (0.0, 4.5) | 0.0 (0.0, 9.0) | 4.0 (0.0, 17.2) |
| Unsaturated fat: saturated fat | 1.5 (1.2, 1.8) | 1.4 (1.1, 1.7) | 1.4 (1.2, 1.8) | 1.5 (1.2, 1.8) | 1.6 (1.3, 1.9) |
| Added sugar (g/day) | 54.8 (36.3, 77.8) | 58.6 (38.4, 85.3) | 55.4 (36.6, 78.8) | 53.8 (35.7, 75.8) | 51.9 (34.8, 72.3) |

Data were presented as frequency (%), mean ± standard deviation or median (interquartile range).

Abbreviation: BMI, body mass index; eGFR, estimated glomerular filtration rate.

**Table S9. Baseline characteristics of the study participants according to categories of the Knuppel PHDI.**

| **Characteristics** | **Total** | **Categories of the Knuppel PHDI** | | | |
| --- | --- | --- | --- | --- | --- |
|  |  | **≤8** | **9** | **10** | **≥11** |
| No. participants | 139165 | 38821 | 40389 | 35307 | 24648 |
| Age (years) | 56.0 (49.0, 62.0) | 55.0 (48.0, 61.0) | 56.0 (49.0, 62.0) | 56.0 (49.0, 62.0) | 57.0 (50.0, 62.0) |
| Sex (male, %) | 65058 (46.7) | 23223 (59.8) | 20105 (49.8) | 14238 (40.3) | 7492 (30.4) |
| Ethnicity (%) |  |  |  |  |  |
| White | 133677 (96.1) | 37496 (96.6) | 38913 (96.3) | 33854 (95.9) | 23414 (95.0) |
| Asian | 1489 (1.1) | 415 (1.1) | 426 (1.1) | 365 (1.0) | 283 (1.1) |
| Black | 2195 (1.6) | 458 (1.2) | 564 (1.4) | 591 (1.7) | 582 (2.4) |
| Other | 1804 (1.3) | 452 (1.2) | 486 (1.2) | 497 (1.4) | 369 (1.5) |
| Education (%) |  |  |  |  |  |
| College or University degree | 68168 (49.0) | 17994 (46.4) | 19456 (48.2) | 17661 (50.0) | 13057 (53.0) |
| Other | 70997 (51.0) | 20827 (53.6) | 20933 (51.8) | 17646 (50.0) | 11591 (47.0) |
| Townsend deprivation index | -2.4 (-3.8, -0.1) | -2.4 (-3.8, -0.1) | -2.5 (-3.8, -0.2) | -2.4 (-3.8, -0.1) | -2.2 (-3.7, 0.2) |
| BMI (%, kg/m2) |  |  |  |  |  |
| Mean (SD) | 26.6 (4.4) | 27.1 (4.5) | 26.7 (4.4) | 26.5 (4.4) | 26.1 (4.4) |
| <18.5 | 730 (0.5) | 146 (0.4) | 177 (0.4) | 219 (0.6) | 188 (0.8) |
| 18.5-24.9 | 54203 (38.9) | 13460 (34.7) | 15300 (37.9) | 14356 (40.7) | 11087 (45.0) |
| 25-29.9 | 58069 (41.7) | 16985 (43.8) | 17243 (42.7) | 14311 (40.5) | 9530 (38.7) |
| ≥30 | 26163 (18.8) | 8230 (21.2) | 7669 (19.0) | 6421 (18.2) | 3843 (15.6) |
| Smoking (n, %) |  |  |  |  |  |
| Never | 80439 (57.8) | 22037 (56.8) | 23559 (58.3) | 20524 (58.1) | 14319 (58.1) |
| Previous | 48296 (34.7) | 13400 (34.5) | 13736 (34.0) | 12405 (35.1) | 8755 (35.5) |
| Current | 10430 (7.5) | 3384 (8.7) | 3094 (7.7) | 2378 (6.7) | 1574 (6.4) |
| Alcohol intake (%) |  |  |  |  |  |
| Never | 7644 (5.5) | 1933 (5.0) | 2047 (5.1) | 2008 (5.7) | 1656 (6.7) |
| Special occasions only | 11979 (8.6) | 3110 (8.0) | 3372 (8.3) | 3100 (8.8) | 2397 (9.7) |
| 1-3 times/month | 14771 (10.6) | 4036 (10.4) | 4193 (10.4) | 3815 (10.8) | 2727 (11.1) |
| 1-2 times/week | 34294 (24.6) | 9514 (24.5) | 10050 (24.9) | 8754 (24.8) | 5976 (24.2) |
| 3-4 times/week | 36856 (26.5) | 10284 (26.5) | 10876 (26.9) | 9277 (26.3) | 6419 (26.0) |
| Daily or almost daily | 33621 (24.2) | 9944 (25.6) | 9851 (24.4) | 8353 (23.7) | 5473 (22.2) |
| Physical activity (%) |  |  |  |  |  |
| Low | 24808 (17.8) | 7846 (20.2) | 7385 (18.3) | 5925 (16.8) | 3652 (14.8) |
| Moderate | 59479 (42.7) | 16632 (42.8) | 17438 (43.2) | 15012 (42.5) | 10397 (42.2) |
| High | 54878 (39.4) | 14343 (36.9) | 15566 (38.5) | 14370 (40.7) | 10599 (43.0) |
| Cancer (%) | 14135 (10.2) | 3575 (9.2) | 3991 (9.9) | 3798 (10.8) | 2771 (11.2) |
| Hypertension (%) | 32652 (23.5) | 9534 (24.6) | 9578 (23.7) | 8171 (23.1) | 5369 (21.8) |
| Diabetes (%) | 5140 (3.7) | 1454 (3.7) | 1493 (3.7) | 1286 (3.6) | 907 (3.7) |
| Cardiovascular disease (%) | 6919 (5.0) | 2089 (5.4) | 2107 (5.2) | 1703 (4.8) | 1020 (4.1) |
| eGFR (mL/min per 1.73 m2) | 93.9 (84.9, 100.9) | 93.6 (84.7, 100.9) | 93.7 (84.6, 100.8) | 93.9 (85.0, 100.8) | 94.4 (85.7, 101.2) |
| Total energy intake (kcal/day) | 2015.4 (1689.0, 2388.0) | 2216.6 (1903.1, 2591.9) | 2052.6 (1747.1, 2408.7) | 1927.3 (1627.7, 2277.2) | 1745.1 (1447.6, 2090.7) |
| Whole grains (g/day) | 215.3 (142.2, 302.6) | 256.0 (169.0, 326.7) | 222.5 (146.0, 311.4) | 198.8 (134.7, 291.0) | 176.0 (116.0, 230.2) |
| Potatoes (g/day) | 87.5 (0.0, 175.0) | 120.0 (45.0, 180.0) | 90.0 (0.0, 175.0) | 65.6 (0.0, 133.8) | 43.8 (0.0, 88.8) |
| Vegetables (g/day) | 171.2 (88.0, 281.6) | 127.5 (67.5, 185.8) | 154.6 (78.3, 253.5) | 210.0 (100.0, 312.4) | 264.1 (179.8, 374.4) |
| Fruits (g/day) | 182.0 (97.5, 294.7) | 100.0 (25.0, 216.0) | 176.0 (100.0, 282.0) | 212.0 (126.0, 321.7) | 246.0 (160.0, 364.5) |
| Dairy (g/day) | 250.0 (160.0, 345.0) | 260.0 (170.0, 361.0) | 253.3 (165.0, 345.0) | 248.0 (160.0, 338.1) | 240.0 (143.8, 330.0) |
| Red meat (g/day) | 46.0 (0.0, 92.0) | 60.0 (30.7, 113.0) | 55.3 (0.0, 100.0) | 32.5 (0.0, 85.7) | 0.0 (0.0, 53.8) |
| Poultry (g/day) | 0.0 (0.0, 65.0) | 32.5 (0.0, 65.0) | 0.0 (0.0, 65.0) | 0.0 (0.0, 43.3) | 0.0 (0.0, 32.5) |
| Eggs (g/day) | 0.0 (0.0, 30.0) | 16.7 (0.0, 50.0) | 0.0 (0.0, 30.0) | 0.0 (0.0, 16.7) | 0.0 (0.0, 0.0) |
| Fish (g/day) | 0.0 (0.0, 50.0) | 0.0 (0.0, 50.0) | 0.0 (0.0, 50.0) | 0.0 (0.0, 50.0) | 20.0 (0.0, 60.0) |
| Legumes | 0.0 (0.0, 11.7) | 0.0 (0.0, 17.5) | 0.0 (0.0, 8.8) | 0.0 (0.0, 8.8) | 0.0 (0.0, 11.7) |
| Soy food (g/day) | 0.0 (0.0, 0.0) | 0.0 (0.0, 0.0) | 0.0 (0.0, 0.0) | 0.0 (0.0, 0.0) | 0.0 (0.0, 0.0) |
| Nuts (g/day) | 0.0 (0.0, 6.7) | 0.0 (0.0, 3.8) | 0.0 (0.0, 6.0) | 0.0 (0.0, 8.1) | 2.0 (0.0, 20.0) |
| Unsaturated fat: saturated fat | 1.5 (1.2, 1.8) | 1.4 (1.2, 1.7) | 1.4 (1.2, 1.7) | 1.5 (1.2, 1.8) | 1.6 (1.3, 2.1) |
| Added sugar (g/day) | 54.8 (36.3, 77.8) | 64.2 (46.5, 87.6) | 57.8 (40.8, 80.3) | 51.2 (33.2, 73.1) | 35.1 (22.5, 59.8) |

Data were presented as frequency (%), mean ± standard deviation or median (interquartile range).

Abbreviation: BMI, body mass index; eGFR, estimated glomerular filtration rate.

**Table S10. Risk of incident CKD according to different genetic risk based on SNPs from Wuttke et al^10^.**

|  |  | **Model 1^a^** | | **Model 2^b^** | | **Model 3^b^** | |
| --- | --- | --- | --- | --- | --- | --- | --- |
|  | **Cases/Total** | **HR (95% CI)** | ***P value*** | **HR (95% CI)** | ***P value*** | **HR (95% CI)** | ***P value*** |
| Low genetic risk | 1557/44019 | REF |  | REF |  | REF |  |
| Intermediate genetic risk | 1862/44018 | 1.211 (1.132-1.295) | <0.001 | 1.208 (1.129-1.292) | <0.001 | 0.981 (0.917-1.051) | 0.589 |
| high genetic risk | 2667/44019 | 1.791 (1.682-1.906) | <0.001 | 1.800 (1.691-1.917) | <0.001 | 1.130 (1.056-1.210) | <0.001 |
| *P* for trend | ‐ | <0.001 |  | <0.001 |  | <0.001 |  |
| 1-point increment in diet index | 6086/132056 | 0.183 (0.158-0.213) | <0.001 | 0.179 (0.153-0.208) | <0.001 | 0.635 (0.537-0.750) | <0.001 |

^a^Model 1 was adjusted for age, sex, ethnicity;

^b^Model 2 was adjusted for model 1 plus Townsend deprivation index, education, hypertension, diabetes, cardiovascular disease, cancer;

^c^Model 3 was adjusted for model 2 plus BMI, eGFR, smoking status, alcohol intake, physical activity, total energy intake.

**Table S11. Associations between the different PHDI and risks of CKD using competing risk models.**

|  | **Cases/Total** | **Model 1^a^** | | **Model 2^b^** | | **Model 3^c^** | |
| --- | --- | --- | --- | --- | --- | --- | --- |
|  |  | **HR (95% CI)** | ***P value*** | **HR (95% CI)** | ***P value*** | **HR (95% CI)** | ***P value*** |
| Stubbendorff PHDI |  |  |  |  |  |  |  |
| ≤18 | 1487/29110 | REF |  | REF |  | REF |  |
| 19-22 | 2297/47472 | 0.912 (0.854-0.973) | 0.006 | 0.922 (0.864-0.984) | 0.015 | 0.960 (0.899-1.020) | 0.200 |
| 23-26 | 1816/40796 | 0.842 (0.785-0.902) | <0.001 | 0.860 (0.803-0.922) | <0.001 | 0.933 (0.870-1.000) | 0.050 |
| ≥27 | 791/21787 | 0.687 (0.630-0.750) | <0.001 | 0.713 (0.653-0.778) | <0.001 | 0.829 (0.759-0.906) | <0.001 |
| *P* for trend |  | <0.001 |  | <0.001 |  | <0.001 |  |
| 1-point increment in diet index | 6391/139165 | 0.974 (0.968-0.979) | <0.001 | 0.976 (0.971-0.982) | <0.001 | 0.987 (0.982-0.993) | <0.001 |
| Colizzi PHDI |  |  |  |  |  |  |  |
| Q1 | 1769/34784 | REF |  | REF |  | REF |  |
| Q2 | 1638/34782 | 0.881 (0.824-0.942) | <0.001 | 0.905 (0.845-0.968) | 0.004 | 0.925 (0.865-0.990) | 0.024 |
| Q3 | 1569/34815 | 0.825 (0.771-0.883) | <0.001 | 0.864 (0.806-0.925) | <0.001 | 0.910 (0.849-0.975) | 0.007 |
| Q4 | 1415/34784 | 0.747 (0.696-0.802) | <0.001 | 0.794 (0.740-0.853) | <0.001 | 0.870 (0.810-0.935) | <0.001 |
| *P* for trend |  | <0.001 |  | <0.001 |  | <0.001 |  |
| 10-point increment in diet index | 6391/139165 | 0.924 (0.908-0.940) | <0.001 | 0.939 (0.923-0.956) | <0.001 | 0.963 (0.946-0.980) | <0.001 |
| Knuppel PHDI |  |  |  |  |  |  |  |
| ≤8 | 1928/38821 | REF |  | REF |  | REF |  |
| 9 | 1887/40389 | 0.919 (0.862-0.979) | 0.009 | 0.924 (0.867-0.985) | 0.015 | 0.949 (0.890-1.010) | 0.110 |
| 10 | 1574/35307 | 0.881 (0.823-0.942) | <0.001 | 0.889 (0.831-0.951) | <0.001 | 0.933 (0.871-1.000) | 0.049 |
| ≥11 | 1002/24648 | 0.808 (0.747-0.873) | <0.001 | 0.822 (0.761-0.889) | <0.001 | 0.888 (0.818-0.963) | 0.004 |
| *P* for trend |  | <0.001 |  | <0.001 |  | 0.004 |  |
| 1-point increment in diet index | 6391/139165 | 0.944 (0.927-0.962) | <0.001 | 0.949 (0.932-0.967) | <0.001 | 0.969 (0.950-0.988) | 0.002 |

^a^Model 1 was adjusted for age, sex, ethnicity;

^b^Model 2 was adjusted for model 1 plus Townsend deprivation index, education, hypertension, diabetes, cardiovascular disease, cancer;

^c^Model 3 was adjusted for model 2 plus BMI, eGFR, smoking status, alcohol intake, physical activity, total energy intake.

**Table S12. Associations between the different PHDI and risks of CKD after excluding individuals diagnosed with CKD within the first three years of follow-up.**

|  | **Cases/Total** | **Model 1^a^** | | **Model 2^b^** | | **Model 3^c^** | |
| --- | --- | --- | --- | --- | --- | --- | --- |
|  |  | **HR (95% CI)** | ***P value*** | **HR (95% CI)** | ***P value*** | **HR (95% CI)** | ***P value*** |
| Stubbendorff PHDI |  |  |  |  |  |  |  |
| ≤18 | 1274/28814 | REF |  | REF |  | REF |  |
| 19-22 | 1986/47048 | 0.917 (0.854-0.984) | 0.016 | 0.926 (0.863-0.993) | 0.032 | 0.963 (0.897-1.034) | 0.294 |
| 23-26 | 1543/40432 | 0.832 (0.772-0.896) | <0.001 | 0.846 (0.785-0.912) | <0.001 | 0.917 (0.851-0.989) | 0.025 |
| ≥27 | 678/21607 | 0.686 (0.625-0.754) | <0.001 | 0.708 (0.644-0.778) | <0.001 | 0.823 (0.748-0.905) | <0.001 |
| *P* for trend |  | <0.001 |  | <0.001 |  | <0.001 |  |
| 1-point increment in diet index | 5481/137901 | 0.973 (0.967-0.979) | <0.001 | 0.975 (0.969-0.981) | <0.001 | 0.986 (0.980-0.993) | <0.001 |
| Colizzi PHDI |  |  |  |  |  |  |  |
| Q1 | 1525/34404 | REF |  | REF |  | REF |  |
| Q2 | 1397/34447 | 0.868 (0.807-0.933) | <0.001 | 0.890 (0.828-0.958) | 0.002 | 0.912 (0.847-0.981) | 0.013 |
| Q3 | 1345/34523 | 0.813 (0.756-0.875) | <0.001 | 0.849 (0.788-0.914) | <0.001 | 0.896 (0.832-0.965) | 0.004 |
| Q4 | 1214/34527 | 0.736 (0.682-0.794) | <0.001 | 0.779 (0.721-0.840) | <0.001 | 0.854 (0.790-0.922) | <0.001 |
| *P* for trend |  | <0.001 |  | <0.001 |  | 0.004 |  |
| 1-point increment in diet index | 5481/137901 | 0.922 (0.905-0.939) | <0.001 | 0.936 (0.919-0.954) | <0.001 | 0.960 (0.942-0.979) | <0.001 |
| Knuppel PHDI |  |  |  |  |  |  |  |
| ≤8 | 1660/38456 | REF |  | REF |  | REF |  |
| 9 | 1630/40041 | 0.923 (0.862-0.988) | 0.022 | 0.928 (0.866-0.994) | 0.033 | 0.953 (0.889-1.021) | 0.171 |
| 10 | 1338/34977 | 0.870 (0.809-0.935) | <0.001 | 0.875 (0.814-0.941) | <0.001 | 0.918 (0.852-0.989) | 0.024 |
| ≥11 | 853/24427 | 0.802 (0.738-0.873) | <0.001 | 0.814 (0.748-0.886) | <0.001 | 0.879 (0.805-0.959) | 0.004 |
| *P* for trend |  | <0.001 |  | <0.001 |  | 0.002 |  |
| 1-point increment in diet index | 5481/137901 | 0.943 (0.924-0.962) | <0.001 | 0.947 (0.929-0.967) | <0.001 | 0.967 (0.946-0.987) | 0.002 |

^a^Model 1 was adjusted for age, sex, ethnicity;

^b^Model 2 was adjusted for model 1 plus Townsend deprivation index, education, hypertension, diabetes, cardiovascular disease, cancer;

^c^Model 3 was adjusted for model 2 plus BMI, eGFR, smoking status, alcohol intake, physical activity, total energy intake.

**Table S13. Associations between the different PHDI and risks of CKD using follow-up phases which began at the time of the completion of the latest dietary questionnaire.**

|  | **Cases/Total** | **Model 1^a^** | | **Model 2^b^** | | **Model 3^c^** | |
| --- | --- | --- | --- | --- | --- | --- | --- |
|  |  | **HR (95% CI)** | ***P value*** | **HR (95% CI)** | ***P value*** | **HR (95% CI)** | ***P value*** |
| Stubbendorff PHDI |  |  |  |  |  |  |  |
| ≤18 | 1307/28924 | REF |  | REF |  | REF |  |
| 19-22 | 2065/47224 | 0.930 (0.867-0.996) | 0.039 | 0.939 (0.876-1.007) | 0.076 | 0.977 (0.911-1.048) | 0.513 |
| 23-26 | 1630/40599 | 0.857 (0.796-0.922) | <0.001 | 0.873 (0.811-0.939) | <0.001 | 0.947 (0.879-1.019) | 0.146 |
| ≥27 | 713/21700 | 0.704 (0.642-0.772) | <0.001 | 0.727 (0.663-0.797) | <0.001 | 0.846 (0.770-0.928) | <0.001 |
| *P* for trend |  | <0.001 |  | <0.001 |  | <0.001 |  |
| 1-point increment in diet index | 5715/138447 | 0.975 (0.969-0.981) | <0.001 | 0.977 (0.971-0.984) | <0.001 | 0.989 (0.982-0.995) | <0.001 |
| Colizzi PHDI |  |  |  |  |  |  |  |
| Q1 | 1612/34618 | REF |  | REF |  | REF |  |
| Q2 | 1467/34596 | 0.860 (0.801-0.924) | <0.001 | 0.885 (0.824-0.950) | <0.001 | 0.903 (0.841-0.969) | 0.005 |
| Q3 | 1390/34629 | 0.794 (0.738-0.853) | <0.001 | 0.830 (0.772-0.892) | <0.001 | 0.872 (0.811-0.938) | <0.001 |
| Q4 | 1246/34604 | 0.713 (0.661-0.768) | <0.001 | 0.755 (0.701-0.814) | <0.001 | 0.823 (0.763-0.887) | <0.001 |
| *P* for trend |  | <0.001 |  | <0.001 |  | 0.004 |  |
| 1-point increment in diet index | 5715/138447 | 0.913 (0.897-0.930) | <0.001 | 0.928 (0.911-0.945) | <0.001 | 0.950 (0.932-0.967) | <0.001 |
| Knuppel PHDI |  |  |  |  |  |  |  |
| ≤8 | 1274/28814 | REF |  | REF |  | REF |  |
| 9 | 1986/47048 | 0.926 (0.865-0.990) | 0.025 | 0.931 (0.871-0.997) | 0.039 | 0.959 (0.896-1.026) | 0.226 |
| 10 | 1543/40432 | 0.891 (0.830-0.957) | 0.002 | 0.897 (0.836-0.964) | 0.003 | 0.946 (0.880-1.017) | 0.133 |
| ≥11 | 678/21607 | 0.830 (0.764-0.901) | <0.001 | 0.844 (0.777-0.916) | <0.001 | 0.917 (0.842-0.999) | 0.048 |
| *P* for trend |  | <0.001 |  | <0.001 |  | 0.041 |  |
| 1-point increment in diet index | 5715/138447 | 0.950 (0.932-0.969) | <0.001 | 0.955 (0.936-0.974) | <0.001 | 0.976 (0.956-0.997) | 0.024 |

^a^Model 1 was adjusted for age, sex, ethnicity;

^b^Model 2 was adjusted for model 1 plus Townsend deprivation index, education, hypertension, diabetes, cardiovascular disease, cancer;

^c^Model 3 was adjusted for model 2 plus BMI, eGFR, smoking status, alcohol intake, physical activity, total energy intake.

**Table S14. Associations between the different PHDI and risks of CKD excluding participants who completed only one the 24-hour dietary questionnaire.**

|  | **Cases/Total** | **Model 1^a^** | | **Model 2^b^** | | **Model 3** | |
| --- | --- | --- | --- | --- | --- | --- | --- |
|  |  | **HR (95% CI)** | ***P value*** | **HR (95% CI)** | ***P value*** | **HR (95% CI)** | ***P value*** |
| Stubbendorff PHDI |  |  |  |  |  |  |  |
| ≤18 | 1024/19976 | REF |  | REF |  | REF |  |
| 19-22 | 1435/30118 | 0.895 (0.825-0.969) | 0.007 | 0.906 (0.836-0.982) | 0.016 | 0.949 (0.875-1.029) | 0.203 |
| 23-26 | 1073/24835 | 0.820 (0.752-0.894) | <0.001 | 0.840 (0.771-0.916) | <0.001 | 0.928 (0.850-1.013) | 0.094 |
| ≥27 | 427/12656 | 0.653 (0.582-0.731) | <0.001 | 0.680 (0.606-0.762) | <0.001 | 0.815 (0.726-0.915) | <0.001 |
| *P* for trend | ‐ | <0.001 |  | <0.001 |  | 0.001 |  |
| 1-point increment in diet index | 3959/87585 | 0.970 (0.963-0.977) | <0.001 | 0.973 (0.966-0.980) | <0.001 | 0.986 (0.979-0.994) | <0.001 |
| Colizzi PHDI |  |  |  |  |  |  |  |
| Q1 | 618/11706 | REF |  | REF |  | REF |  |
| Q2 | 932/19499 | 0.856 (0.774-0.948) | 0.003 | 0.878 (0.793-0.972) | 0.012 | 0.913 (0.824-1.011) | 0.079 |
| Q3 | 1162/25544 | 0.799 (0.724-0.881) | <0.001 | 0.829 (0.752-0.915) | <0.001 | 0.889 (0.806-0.982) | 0.020 |
| Q4 | 1247/30836 | 0.715 (0.649-0.788) | <0.001 | 0.755 (0.685-0.832) | <0.001 | 0.844 (0.764-0.931) | <0.001 |
| *P* for trend | ‐ | <0.001 |  | <0.001 |  | <0.001 |  |
| 1-point increment in diet index | 3959/87585 | 0.914 (0892-0.935) | <0.001 | 0.927 (0.905-0.949) | <0.001 | 0.956 (0.933-0.979) | <0.001 |
| Knuppel PHDI |  |  |  |  |  |  |  |
| ≤9 | 1290/26028 | REF |  | REF |  | REF |  |
| 10 | 1187/25653 | 0.916 (0.847-0.992) | 0.030 | 0.921 (0.851-0.997) | 0.041 | 0.955 (0.881-1.034) | 0.254 |
| 11 | 936/21521 | 0.866 (0.795-0.943) | <0.001 | 0.880 (0.808-0.958) | 0.003 | 0.930 (0.853-1.015) | 0.103 |
| ≥12 | 546/14383 | 0.767 (0.693-0.850) | <0.001 | 0.783 (0.706-0.867) | <0.001 | 0.860 (0.773-0.956) | 0.005 |
| *P* for trend | ‐ | <0.001 |  | <0.001 |  | 0.005 |  |
| 1-point increment in diet index | 3959/87585 | 0.933 (0.911-0.956) | <0.001 | 0.940 (0.917-0.962) | <0.001 | 0.962 (0.938-0.987) | 0.003 |

^a^Model 1 was adjusted for age, sex, ethnicity;

^b^Model 2 was adjusted for model 1 plus Townsend deprivation index, education, hypertension, diabetes, cardiovascular disease, cancer;

^c^Model 3 was adjusted for model 2 plus BMI, eGFR, smoking status, alcohol intake, physical activity, total energy intake.

**Table S15. Mediating effects of BMI, CVD, diabetes and hypertension in the associations between the different PHDIs and risks of CKD.**

|  | **Mediation proportion^a^ (95%CI), %** | | | |
| --- | --- | --- | --- | --- |
|  | **BMI** | **Hypertension** | **CVD** | **Diabetes** |
| Stubbendorff PHDI | 18.50 (12.10-31.00) | 0.55 (-1.78-3.00) | 0.02 (-0.96-1.00) | 1.59 (-4.15-0.00) |
| Colizzi PHDI | 16.50 (10.80-30.00) | 0.65 (-2.21-4.00) | 0.37 (-0.65-2.00) | -0.14 (-2.25-2.00) |
| Knuppel PHDI | 11.92 (7.00-30.00) | 1.17 (-2.14-7.00) | -0.18 (-4.10-4.00) | -0.98 (-18.82-5.00) |

^a^Models were adjusted for age, sex, ethnicity, Townsend deprivation index, education, smoking status, alcohol intake, physical activity, total energy intake, and BMI, CVD, diabetes and hypertension when these were not considered the potential mediator.

Abbreviation: BMI, body mass index; CVD, cardiovascular disease.

**Table S16. Subgroup analysis of associations between the Stubbendorff PHDI and risks of CKD.**

| **Covariates** | **No.** | **Categories of Stubbendorff PHDI** | | | | | | ***P* value** | ***P* for interaction** |
| --- | --- | --- | --- | --- | --- | --- | --- | --- | --- |
|  |  | **≤18** | **19-22** |  | **23-26** |  | **≥27** |  |  |
|  |  |  | **HR^a^ (95% CI)** | ***P* value** | **HR (95% CI)** | ***P* value** | **HR (95% CI)** |  |  |
| Age |  |  |  |  |  |  |  |  | 0.423 |
| <60 | 89012 | REF | 0.942 (0.851-1.043) | 0.248 | 0.867 (0.779-0.965) | 0.009 | 0.743 (0.651-0.848) | <0.001 |  |
| ≥60 | 50153 | REF | 0.889 (0.816-0.968) | 0.007 | 0.811 (0.742-0.887) | <0.001 | 0.641 (0.572-0.718) | <0.001 |  |
| Sex |  |  |  |  |  |  |  |  | 0.745 |
| Female | 74107 | REF | 0.935 (0.844-1.036) | 0.199 | 0.883 (0.795-0.980) | 0.019 | 0.710 (0.626-0.804) | <0.001 |  |
| Male | 65058 | REF | 0.990 (0.910-1.078) | 0.817 | 0.917 (0.836-1.005) | 0.064 | 0.778 (0.689-0.879) | <0.001 |  |
| Education |  |  |  |  |  |  |  |  | 0.784 |
| College or University degree | 68168 | REF | 0.935 (0.841-1.040) | 0.216 | 0.889 (0.798-0.991) | 0.034 | 0.707 (0.620-0.805) | <0.001 |  |
| Other | 70997 | REF | 0.973 (0.895-1.057) | 0.511 | 0.890 (0.814-0.973) | 0.011 | 0.762 (0.678-0.856) | <0.001 |  |
| BMI |  |  |  |  |  |  |  |  | 0.561 |
| <25 | 54933 | REF | 0.912 (0.800-1.041) | 0.172 | 0.854 (0.747-0.976) | 0.021 | 0.704 (0.602-0.823) | <0.001 |  |
| ≥25 | 84232 | REF | 0.982 (0.911-1.059) | 0.636 | 0.928 (0.857-1.006) | 0.069 | 0.802 (0.722-0.891) | <0.001 |  |
| Hypertension |  |  |  |  |  |  |  |  | 0.101 |
| No | 106513 | REF | 0.984 (0.903-1.073) | 0.721 | 0.865 (0.790-0.948) | 0.002 | 0.759 (0.679-0.848) | <0.001 |  |
| Yes | 32652 | REF | 0.912 (0.826-1.008) | 0.072 | 0.924 (0.833-1.026) | 0.139 | 0.691 (0.602-0.793) | <0.001 |  |
| Diabetes |  |  |  |  |  |  |  |  | 0.355 |
| No | 134025 | REF | 0.960 (0.896-1.029) | 0.248 | 0.878 (0.817-0.944) | <0.001 | 0.717 (0.655-0.786) | <0.001 |  |
| Yes | 5140 | REF | 0.800 (0.653-0.981) | 0.032 | 0.747 (0.605-0.923) | 0.007 | 0.597 (0.455-0.785) | <0.001 |  |
| Cardiovascular disease |  |  |  |  |  |  |  |  | 0.193 |
| No | 132246 | REF | 0.970 (0.904-1.041) | 0.399 | 0.896 (0.832-0.964) | 0.003 | 0.722 (0.658-0.792) | <0.001 |  |
| Yes | 6919 | REF | 0.791 (0.663-0.943) | 0.009 | 0.766 (0.633-0.927) | 0.006 | 0.665 (0.523-0.844) | 0.001 |  |
| Cancer |  |  |  |  |  |  |  |  | 0.868 |
| No | 125030 | REF | 0.935 (0.872-1.004) | 0.063 | 0.860 (0.799-0.926) | <0.001 | 0.705 (0.642-0.773) | <0.001 |  |
| Yes | 14135 | REF | 0.995 (0.835-1.186) | 0.959 | 0.909 (0.757-1.092) | 0.309 | 0.698 (0.554-0.879) | 0.002 |  |
| Ethnic |  |  |  |  |  |  |  |  | 0.336 |
| White | 133677 | REF | 0.951 (0.890-1.016) | 0.135 | 0.872 (0.813-0.935) | <0.001 | 0.696 (0.637-0.760) | <0.001 |  |
| Asian | 1489 | REF | 0.876 (0.462-1.663) | 0.687 | 0.931 (0.491-1.765) | 0.826 | 0.733 (0.343-1.567) | 0.424 |  |
| Black | 2195 | REF | 0.477 (0.236-0.965) | 0.04 | 0.536 (0.284-1.012) | 0.054 | 0.754 (0.407-1.396) | 0.368 |  |
| Other | 1804 | REF | 1.197 (0.594-2.414) | 0.615 | 1.104 (0.538-2.265) | 0.788 | 1.073 (0.502-2.290) | 0.856 |  |
| Townsend deprivation index |  |  |  |  |  |  |  |  | 0.709 |
| Above median | 69737 | REF | 0.939 (0.855-1.032) | 0.194 | 0.895 (0.812-0.987) | 0.026 | 0.707 (0.627-0.797) | <0.001 |  |
| Others | 69428 | REF | 0.952 (0.870-1.042) | 0.289 | 0.846 (0.768-0.931) | 0.001 | 0.709 (0.627-0.803) | <0.001 |  |
| Smoking status |  |  |  |  |  |  |  |  | 0.234 |
| Never | 80439 | REF | 1.003 (0.914-1.101) | 0.953 | 0.918 (0.833-1.011) | 0.084 | 0.773 (0.686-0.871) | <0.001 |  |
| Previous | 48296 | REF | 0.872 (0.788-0.965) | 0.008 | 0.834 (0.750-0.927) | 0.001 | 0.638 (0.558-0.731) | <0.001 |  |
| Current | 10430 | REF | 1.014 (0.820-1.254) | 0.897 | 0.780 (0.609-1.000) | 0.05 | 0.689 (0.490-0.969) | 0.032 |  |
| Alcohol consumption status |  |  |  |  |  |  |  |  | 0.759 |
| Never | 7644 | REF | 0.965 (0.753-1.237) | 0.781 | 0.802 (0.622-1.035) | 0.091 | 0.650 (0.483-0.874) | 0.004 |  |
| Special occasions only | 11979 | REF | 0.811 (0.664-0.990) | 0.039 | 0.741 (0.602-0.911) | 0.004 | 0.578 (0.449-0.746) | <0.001 |  |
| 1-3 times/month | 14771 | REF | 0.932 (0.762-1.141) | 0.496 | 0.880 (0.714-1.084) | 0.229 | 0.790 (0.616-1.014) | 0.064 |  |
| 1-2 times/week | 34294 | REF | 0.884 (0.774-1.010) | 0.069 | 0.836 (0.728-0.960) | 0.011 | 0.665 (0.557-0.794) | <0.001 |  |
| 3-4 times/week | 36856 | REF | 1.086 (0.946-1.248) | 0.24 | 0.973 (0.841-1.126) | 0.716 | 0.744 (0.618-0.896) | 0.002 |  |
| Daily or almost daily | 33621 | REF | 0.943 (0.828-1.073) | 0.372 | 0.859 (0.747-0.987) | 0.032 | 0.706 (0.587-0.848) | <0.001 |  |
| Physical activity |  |  |  |  |  |  |  |  | 0.535 |
| Low | 24808 | REF | 0.944 (0.824-1.083) | 0.413 | 0.941 (0.813-1.089) | 0.414 | 0.688 (0.558-0.848) | <0.001 |  |
| Moderate | 59479 | REF | 0.938 (0.849-1.036) | 0.204 | 0.817 (0.734-0.908) | <0.001 | 0.726 (0.637-0.829) | <0.001 |  |
| High | 54878 | REF | 0.974 (0.871-1.089) | 0.644 | 0.919 (0.819-1.031) | 0.149 | 0.722 (0.629-0.830) | <0.001 |  |

^a^Adjusted for age, sex, ethnicity, Townsend deprivation index, education, hypertension, diabetes, cardiovascular disease, cancer, BMI, eGFR, smoking status, alcohol intake, physical activity, total energy intake.

**Table S17. Subgroup analysis of associations between the Colizzi PHDI and risks of CKD.**

| **Covariates** | **No.** | **Categories of Colizzi PHDI** | | | | | | ***P* value** | ***P* for interaction** |
| --- | --- | --- | --- | --- | --- | --- | --- | --- | --- |
|  |  | **Q1** | **Q2** |  | **Q3** |  | **Q4** |  |  |
|  |  |  | **HR (95% CI)** | ***P* value** | **HR (95% CI)** | ***P* value** | **HR (95% CI)** |  |  |
| Age |  |  |  |  |  |  |  |  | 0.239 |
| <60 | 89012 | REF | 0.886 (0.798-0.984) | 0.024 | 0.868 (0.781-0.964) | 0.009 | 0.798 (0.716-0.889) | <0.001 |  |
| ≥60 | 50153 | REF | 0.870 (0.797-0.950) | 0.002 | 0.790 (0.723-0.864) | <0.001 | 0.701 (0.640-0.768) | <0.001 |  |
| Sex |  |  |  |  |  |  |  |  | 0.395 |
| Female | 74107 | REF | 0.886 (0.799-0.982) | 0.021 | 0.872 (0.788-0.966) | 0.009 | 0.779 (0.702-0.863) | <0.001 |  |
| Male | 65058 | REF | 0.982 (0.898-1.073) | 0.683 | 0.924 (0.843-1.013) | 0.091 | 0.865 (0.785-0.952) | 0.003 |  |
| Education |  |  |  |  |  |  |  |  | 0.348 |
| College or University degree | 68168 | REF | 0.905 (0.811-1.009) | 0.072 | 0.877 (0.788-0.977) | 0.017 | 0.770 (0.691-0.858) | <0.001 |  |
| Other | 70997 | REF | 0.956 (0.878-1.041) | 0.3 | 0.918 (0.840-1.002) | 0.056 | 0.878 (0.800-0.963) | 0.006 |  |
| BMI |  |  |  |  |  |  |  |  | 0.214 |
| <25 | 54933 | REF | 0.856 (0.749-0.979) | 0.023 | 0.846 (0.742-0.964) | 0.012 | 0.754 (0.661-0.860) | <0.001 |  |
| ≥25 | 84232 | REF | 0.972 (0.899-1.050) | 0.467 | 0.936 (0.865-1.014) | 0.106 | 0.883 (0.813-0.960) | 0.003 |  |
| Hypertension |  |  |  |  |  |  |  |  | 0.617 |
| No | 106513 | REF | 0.933 (0.854-1.020) | 0.13 | 0.897 (0.820-0.982) | 0.018 | 0.843 (0.769-0.923) | <0.001 |  |
| Yes | 32652 | REF | 0.925 (0.835-1.024) | 0.134 | 0.885 (0.797-0.982) | 0.021 | 0.770 (0.690-0.860) | <0.001 |  |
| Diabetes |  |  |  |  |  |  |  |  | 0.292 |
| No | 134025 | REF | 0.938 (0.874-1.008) | 0.081 | 0.900 (0.838-0.967) | 0.004 | 0.818 (0.760-0.880) | <0.001 |  |
| Yes | 5140 | REF | 0.817 (0.666-1.001) | 0.051 | 0.758 (0.614-0.936) | 0.01 | 0.668 (0.534-0.836) | <0.001 |  |
| Cardiovascular disease |  |  |  |  |  |  |  |  | 0.177 |
| No | 132246 | REF | 0.904 (0.841-0.972) | 0.006 | 0.882 (0.820-0.948) | 0.001 | 0.796 (0.739-0.858) | <0.001 |  |
| Yes | 6919 | REF | 1.109 (0.923-1.333) | 0.269 | 0.894 (0.737-1.085) | 0.256 | 0.852 (0.697-1.041) | 0.117 |  |
| Cancer |  |  |  |  |  |  |  |  | 0.832 |
| No | 125030 | REF | 0.918 (0.854-0.987) | 0.021 | 0.865 (0.804-0.931) | <0.001 | 0.789 (0.732-0.851) | <0.001 |  |
| Yes | 14135 | REF | 0.924 (0.772-1.107) | 0.39 | 0.934 (0.780-1.117) | 0.452 | 0.784 (0.651-0.944) | 0.01 |  |
| Ethnic |  |  |  |  |  |  |  |  | 0.084 |
| White | 133677 | REF | 0.912 (0.852-0.977) | 0.009 | 0.867 (0.809-0.930) | <0.001 | 0.788 (0.734-0.846) | <0.001 |  |
| Asian | 1489 | REF | 1.632 (0.915-2.909) | 0.097 | 1.429 (0.771-2.649) | 0.257 | 1.417 (0.718-2.799) | 0.315 |  |
| Black | 2195 | REF | 0.764 (0.401-1.454) | 0.412 | 0.844 (0.464-1.536) | 0.58 | 0.981 (0.563-1.707) | 0.945 |  |
| Other | 1804 | REF | 1.291 (0.709-2.350) | 0.404 | 1.428 (0.793-2.572) | 0.235 | 0.506 (0.237-1.081) | 0.079 |  |
| Townsend deprivation index |  |  |  |  |  |  |  |  | 0.958 |
| Above median | 69737 | REF | 0.937 (0.852-1.032) | 0.187 | 0.891 (0.808-0.982) | 0.02 | 0.797 (0.721-0.880) | <0.001 |  |
| Others | 69428 | REF | 0.906 (0.825-0.995) | 0.04 | 0.865 (0.786-0.951) | 0.003 | 0.787 (0.714-0.868) | <0.001 |  |
| Smoking status |  |  |  |  |  |  |  |  | 0.573 |
| Never | 80439 | REF | 0.949 (0.863-1.043) | 0.274 | 0.874 (0.794-0.963) | 0.006 | 0.823 (0.747-0.907) | <0.001 |  |
| Previous | 48296 | REF | 0.925 (0.832-1.027) | 0.145 | 0.886 (0.797-0.984) | 0.024 | 0.762 (0.682-0.850) | <0.001 |  |
| Current | 10430 | REF | 0.782 (0.618-0.989) | 0.04 | 0.913 (0.722-1.154) | 0.445 | 0.801 (0.616-1.041) | 0.097 |  |
| Alcohol consumption status |  |  |  |  |  |  |  |  | 0.884 |
| Never | 7644 | REF | 0.952 (0.749-1.211) | 0.689 | 0.831 (0.650-1.063) | 0.141 | 0.687 (0.532-0.888) | 0.004 |  |
| Special occasions only | 11979 | REF | 0.913 (0.747-1.115) | 0.371 | 0.766 (0.620-0.947) | 0.014 | 0.794 (0.644-0.978) | 0.03 |  |
| 1-3 times/month | 14771 | REF | 0.989 (0.805-1.215) | 0.916 | 0.967 (0.786-1.190) | 0.752 | 0.859 (0.695-1.062) | 0.16 |  |
| 1-2 times/week | 34294 | REF | 0.937 (0.815-1.077) | 0.36 | 0.970 (0.845-1.114) | 0.67 | 0.794 (0.687-0.919) | 0.002 |  |
| 3-4 times/week | 36856 | REF | 0.910 (0.790-1.049) | 0.193 | 0.846 (0.734-0.976) | 0.022 | 0.818 (0.709-0.944) | 0.006 |  |
| Daily or almost daily | 33621 | REF | 0.891 (0.777-1.021) | 0.097 | 0.859 (0.748-0.986) | 0.031 | 0.776 (0.672-0.897) | 0.001 |  |
| Physical activity |  |  |  |  |  |  |  |  | 0.165 |
| Low | 24808 | REF | 0.933 (0.808-1.077) | 0.345 | 0.947 (0.817-1.098) | 0.471 | 0.859 (0.735-1.006) | 0.059 |  |
| Moderate | 59479 | REF | 0.932 (0.841-1.033) | 0.181 | 0.881 (0.794-0.978) | 0.017 | 0.726 (0.651-0.810) | <0.001 |  |
| High | 54878 | REF | 0.912 (0.814-1.022) | 0.112 | 0.856 (0.764-0.959) | 0.007 | 0.855 (0.764-0.957) | 0.006 |  |

^a^Adjusted for age, sex, ethnicity, Townsend deprivation index, education, hypertension, diabetes, cardiovascular disease, cancer, BMI, eGFR, smoking status, alcohol intake, physical activity, total energy intake.

**Table S18. Subgroup analysis of associations between the Knuppel PHDI and risks of CKD.**

| **Covariates** | **No.** | **Categories of Knuppel PHDI** | | | | | | ***P* value** | ***P* for interaction** |
| --- | --- | --- | --- | --- | --- | --- | --- | --- | --- |
|  |  | **≤9** | **10** |  | **11** |  | **≥12** |  |  |
|  |  |  | **HR^a^ (95% CI)** | ***P* value** | **HR (95% CI)** | ***P* value** | **HR (95% CI)** |  |  |
| Age |  |  |  |  |  |  |  |  | 0.046 |
| <60 | 89012 | REF | 0.963 (0.871-1.064) | 0.455 | 0.962 (0.867-1.067) | 0.459 | 0.861 (0.764-0.971) | 0.015 |  |
| ≥60 | 50153 | REF | 0.882 (0.812-0.957) | 0.003 | 0.801 (0.735-0.874) | <0.001 | 0.738 (0.668-0.814) | <0.001 |  |
| Sex |  |  |  |  |  |  |  |  | 0.664 |
| Female | 74107 | REF | 0.958 (0.864-1.061) | 0.409 | 0.958 (0.865-1.061) | 0.413 | 0.865 (0.775-0.965) | 0.009 |  |
| Male | 65058 | REF | 0.976 (0.899-1.058) | 0.55 | 0.934 (0.853-1.023) | 0.143 | 0.933 (0.832-1.046) | 0.236 |  |
| Education |  |  |  |  |  |  |  |  | 0.853 |
| College or University degree | 68168 | REF | 0.958 (0.866-1.059) | 0.399 | 0.939 (0.847-1.042) | 0.235 | 0.845 (0.752-0.949) | 0.004 |  |
| Other | 70997 | REF | 0.939 (0.866-1.020) | 0.134 | 0.885 (0.811-0.965) | 0.006 | 0.830 (0.750-0.918) | <0.001 |  |
| BMI |  |  |  |  |  |  |  |  | 0.718 |
| <25 | 54933 | REF | 0.949 (0.838-1.074) | 0.406 | 0.867 (0.762-0.986) | 0.029 | 0.814 (0.707-0.936) | 0.004 |  |
| ≥25 | 84232 | REF | 0.958 (0.890-1.032) | 0.257 | 0.948 (0.877-1.025) | 0.178 | 0.886 (0.808-0.970) | 0.009 |  |
| Hypertension |  |  |  |  |  |  |  |  | 0.101 |
| No | 106513 | REF | 0.958 (0.880-1.042) | 0.316 | 0.948 (0.869-1.035) | 0.236 | 0.903 (0.819-0.996) | 0.042 |  |
| Yes | 32652 | REF | 0.939 (0.853-1.034) | 0.201 | 0.858 (0.775-0.951) | 0.004 | 0.757 (0.669-0.855) | <0.001 |  |
| Diabetes |  |  |  |  |  |  |  |  | 0.418 |
| No | 134025 | REF | 0.944 (0.883-1.009) | 0.091 | 0.897 (0.836-0.963) | 0.003 | 0.834 (0.769-0.903) | <0.001 |  |
| Yes | 5140 | REF | 0.925 (0.759-1.128) | 0.442 | 0.912 (0.742-1.122) | 0.383 | 0.684 (0.533-0.879) | 0.003 |  |
| CVD |  |  |  |  |  |  |  |  | 0.11 |
| No | 132246 | REF | 0.945 (0.883-1.012) | 0.106 | 0.930 (0.866-0.998) | 0.044 | 0.846 (0.780-0.917) | <0.001 |  |
| Yes | 6919 | REF | 0.933 (0.787-1.105) | 0.419 | 0.737 (0.609-0.893) | 0.002 | 0.762 (0.608-0.954) | 0.018 |  |
| Cancer |  |  |  |  |  |  |  |  | 0.903 |
| No | 125030 | REF | 0.939 (0.877-1.005) | 0.071 | 0.898 (0.836-0.965) | 0.004 | 0.812 (0.747-0.882) | <0.001 |  |
| Yes | 14135 | REF | 0.929 (0.783-1.102) | 0.396 | 0.839 (0.703-1.002) | 0.052 | 0.799 (0.656-0.974) | 0.026 |  |
| Ethnic |  |  |  |  |  |  |  |  | 0.231 |
| White | 133677 | REF | 0.940 (0.881-1.002) | 0.059 | 0.894 (0.835-0.956) | 0.001 | 0.807 (0.746-0.873) | <0.001 |  |
| Asian | 1489 | REF | 0.769 (0.391-1.513) | 0.446 | 1.400 (0.763-2.571) | 0.278 | 1.563 (0.834-2.928) | 0.163 |  |
| Black | 2195 | REF | 0.813 (0.444-1.488) | 0.502 | 0.816 (0.449-1.485) | 0.506 | 0.907 (0.505-1.629) | 0.743 |  |
| Other | 1804 | REF | 1.427 (0.786-2.591) | 0.243 | 0.922 (0.480-1.772) | 0.807 | 1.010 (0.509-2.004) | 0.977 |  |
| Townsend deprivation index |  |  |  |  |  |  |  |  | 0.317 |
| Above median | 69737 | REF | 0.973 (0.888-1.065) | 0.547 | 0.892 (0.811-0.982) | 0.019 | 0.788 (0.707-0.879) | <0.001 |  |
| Others | 69428 | REF | 0.912 (0.834-0.996) | 0.041 | 0.901 (0.822-0.989) | 0.028 | 0.852 (0.765-0.948) | 0.003 |  |
| Smoking status |  |  |  |  |  |  |  |  | 0.007 |
| Never | 80439 | REF | 0.972 (0.889-1.063) | 0.537 | 0.936 (0.852-1.029) | 0.17 | 0.935 (0.842-1.038) | 0.206 |  |
| Previous | 48296 | REF | 0.891 (0.808-0.983) | 0.022 | 0.849 (0.767-0.941) | 0.002 | 0.673 (0.595-0.761) | <0.001 |  |
| Current | 10430 | REF | 1.055 (0.847-1.315) | 0.633 | 0.909 (0.710-1.163) | 0.447 | 0.909 (0.686-1.205) | 0.509 |  |
| Alcohol consumption status |  |  |  |  |  |  |  |  | 0.926 |
| Never | 7644 | REF | 1.009 (0.794-1.283) | 0.94 | 1.014 (0.797-1.290) | 0.912 | 0.729 (0.552-0.962) | 0.026 |  |
| Special occasions only | 11979 | REF | 0.981 (0.807-1.193) | 0.851 | 0.871 (0.709-1.070) | 0.189 | 0.801 (0.638-1.005) | 0.056 |  |
| 1-3 times/month | 14771 | REF | 1.005 (0.825-1.223) | 0.963 | 0.989 (0.808-1.211) | 0.916 | 0.882 (0.701-1.110) | 0.284 |  |
| 1-2 times/week | 34294 | REF | 0.869 (0.764-0.988) | 0.032 | 0.802 (0.699-0.919) | 0.002 | 0.793 (0.679-0.925) | 0.003 |  |
| 3-4 times/week | 36856 | REF | 0.953 (0.836-1.087) | 0.476 | 0.907 (0.790-1.042) | 0.169 | 0.805 (0.686-0.945) | 0.008 |  |
| Daily or almost daily | 33621 | REF | 0.938 (0.824-1.067) | 0.331 | 0.901 (0.787-1.033) | 0.136 | 0.830 (0.707-0.974) | 0.022 |  |
| Physical activity |  |  |  |  |  |  |  |  | 0.947 |
| Low | 24808 | REF | 0.944 (0.825-1.081) | 0.405 | 0.872 (0.753-1.010) | 0.067 | 0.788 (0.659-0.942) | 0.009 |  |
| Moderate | 59479 | REF | 0.935 (0.848-1.030) | 0.172 | 0.880 (0.794-0.976) | 0.015 | 0.815 (0.724-0.916) | 0.001 |  |
| High | 54878 | REF | 0.958 (0.861-1.066) | 0.432 | 0.948 (0.849-1.057) | 0.335 | 0.862 (0.762-0.974) | 0.017 |  |

^a^Adjusted for age, sex, ethnicity, Townsend deprivation index, education, hypertension, diabetes, cardiovascular disease, cancer, BMI, eGFR, smoking status, alcohol intake, physical activity, total energy intake.

**Table S19. Risk of incident CKD according to different genetic risk base on the SNPs from Yu et al^11^.**

|  | **Cases/Total** | **Model 1^a^** | | **Model 2^b^** | | **Model 3^c^** | |
| --- | --- | --- | --- | --- | --- | --- | --- |
|  |  | **HR (95% CI)** | ***P value*** | **HR (95% CI)** | ***P value*** | **HR (95% CI)** | ***P value*** |
| Low genetic risk | 1738/44016 | REF |  | REF |  | REF |  |
| Intermediate genetic risk | 1940/44021 | 1.132 (1.061-1.207) | <0.001 | 1.130 (1.059-1.206) | <0.001 | 0.999 (0.936-1.066) | 0.973 |
| high genetic risk | 2408/44019 | 1.409 (1.325-1.499) | <0.001 | 1.422 (1.337-1.512) | <0.001 | 1.110 (1.043-1.183) | <0.001 |
| *P* for trend | ‐ | <0.001 |  | <0.001 |  | <0.001 |  |
| 1-point increment in diet index | 6086/132056 | 0.006 (0.003-0.013) | <0.001 | 0.005 (0.002-0.012) | <0.001 | 0.204 (0.088-0.476) | <0.001 |

^a^Model 1 was adjusted for age, sex, ethnicity;

^b^Model 2 was adjusted for model 1 plus Townsend deprivation index, education, hypertension, diabetes, cardiovascular disease, cancer;

^c^Model 3 was adjusted for model 2 plus BMI, eGFR, smoking status, alcohol intake, physical activity, total energy intake.

**Table S20. Baseline characteristics of the study participants according to categories of the Stubbendorff PHDI after imputation.**

| **Characteristics** | **Total** | **Categories of the Stubbendorff PHDI** | | | |
| --- | --- | --- | --- | --- | --- |
|  |  | **≤18** | **19-22** | **23-26** | **≥27** |
| No. participants | 193584 | 41074 | 66701 | 56483 | 29326 |
| Age (years) | 57.0 (50.0, 62.0) | 56.0 (49.0, 62.0) | 57.0 (50.0, 62.0) | 57.0 (50.0, 62.0) | 57.0 (50.0, 62.0) |
| Sex (male, %) | 86676 (44.8) | 22099 (53.8) | 30832 (46.2) | 23012 (40.7) | 10733 (36.6) |
| Ethnicity (%) |  |  |  |  |  |
| White | 185724 (95.9) | 39959 (97.3) | 64375 (96.5) | 53962 (95.5) | 27428 (93.5) |
| Asian | 2264 (1.2) | 386 (0.9) | 766 (1.1) | 684 (1.2) | 428 (1.5) |
| Black | 3099 (1.6) | 336 (0.8) | 768 (1.2) | 1067 (1.9) | 928 (3.2) |
| Other | 2497 (1.3) | 393 (1.0) | 792 (1.2) | 770 (1.4) | 542 (1.8) |
| Education (%) |  |  |  |  |  |
| College or University degree | 90267 (46.6) | 16905 (41.2) | 29893 (44.8) | 27629 (48.9) | 15840 (54.0) |
| Other | 103317 (53.4) | 24169 (58.8) | 36808 (55.2) | 28854 (51.1) | 13486 (46.0) |
| Townsend deprivation index | -2.3 (-3.7, 0.0) | -2.4 (-3.8, 0.0) | -2.4 (-3.8, -0.1) | -2.3 (-3.7, 0.0) | -2.0 (-3.6, 0.5) |
| BMI (%, kg/m2) |  |  |  |  |  |
| Mean (SD) | 26.8 (4.6) | 27.5 (4.6) | 27.1 (4.6) | 26.6 (4.5) | 25.8 (4.3) |
| <18.5 | 1009 (0.5) | 150 (0.4) | 275 (0.4) | 306 (0.5) | 278 (0.9) |
| 18.5-24.9 | 72652 (37.5) | 12817 (31.2) | 23335 (35.0) | 22585 (40.0) | 13915 (47.4) |
| 25-29.9 | 80728 (41.7) | 18072 (44.0) | 28656 (43.0) | 23121 (40.9) | 10879 (37.1) |
| ≥30 | 39195 (20.2) | 10035 (24.4) | 14435 (21.6) | 10471 (18.5) | 4254 (14.5) |
| Smoking (n, %) |  |  |  |  |  |
| Never | 110174 (56.9) | 22277 (54.2) | 37693 (56.5) | 32899 (58.2) | 17305 (59.0) |
| Previous | 68319 (35.3) | 14291 (34.8) | 23667 (35.5) | 19933 (35.3) | 10428 (35.6) |
| Current | 15091 (7.8) | 4506 (11.0) | 5341 (8.0) | 3651 (6.5) | 1593 (5.4) |
| Alcohol intake (%) |  |  |  |  |  |
| Never | 11788 (6.1) | 2173 (5.3) | 3719 (5.6) | 3571 (6.3) | 2325 (7.9) |
| Special occasions only | 18668 (9.6) | 3620 (8.8) | 6241 (9.4) | 5584 (9.9) | 3223 (11.0) |
| 1-3 times/month | 21240 (11.0) | 4446 (10.8) | 7190 (10.8) | 6212 (11.0) | 3392 (11.6) |
| 1-2 times/week | 48329 (25.0) | 10026 (24.4) | 16806 (25.2) | 14240 (25.2) | 7257 (24.7) |
| 3-4 times/week | 49117 (25.4) | 10272 (25.0) | 17111 (25.7) | 14417 (25.5) | 7317 (25.0) |
| Daily or almost daily | 44442 (23.0) | 10537 (25.7) | 15634 (23.4) | 12459 (22.1) | 5812 (19.8) |
| Physical activity (%) |  |  |  |  |  |
| Low | 34684 (17.9) | 8812 (21.5) | 12413 (18.6) | 9345 (16.5) | 4114 (14.0) |
| Moderate | 82085 (42.4) | 17317 (42.2) | 28768 (43.1) | 23841 (42.2) | 12159 (41.5) |
| High | 76815 (39.7) | 14945 (36.4) | 25520 (38.3) | 23297 (41.2) | 13053 (44.5) |
| Cancer (%) | 20425 (10.6) | 4138 (10.1) | 7034 (10.5) | 6095 (10.8) | 3158 (10.8) |
| Hypertension (%) | 48211 (24.9) | 10801 (26.3) | 17108 (25.6) | 13600 (24.1) | 6702 (22.9) |
| Diabetes (%) | 7925 (4.1) | 1608 (3.9) | 2753 (4.1) | 2371 (4.2) | 1193 (4.1) |
| Cardiovascular disease (%) | 10717 (5.5) | 2411 (5.9) | 3901 (5.8) | 2962 (5.2) | 1443 (4.9) |
| eGFR (mL/min per 1.73 m^2^) | 93.5 (84.4, 100.4) | 93.0 (83.9, 100.3) | 93.0 (83.8, 99.9) | 93.7 (84.7, 100.5) | 94.9 (86.4, 101.7) |
| Total energy intake(kcal/day) | 1996.8 (1669.9, 2371.6) | 2103.2 (1769.8, 2487.4) | 2006.1 (1679.0, 2372.2) | 1946.1 (1624.0, 2310.9) | 1932.8 (1612.4, 2310.9) |
| Vegetables (g/day) | 170.0 (84.9, 280.8) | 127.7 (65.0, 203.0) | 153.2 (73.6, 251.0) | 189.5 (93.9, 305.2) | 263.1 (155.0, 388.0) |
| Fruits (g/day) | 180.0 (95.0, 293.0) | 95.0 (0.0, 180.8) | 164.0 (85.0, 270.0) | 212.5 (120.0, 324.8) | 265.0 (180.0, 380.0) |
| Unsaturated oils (g/day) | 0.0 (0.0, 10.0) | 0.0 (0.0, 7.0) | 0.0 (0.0, 10.0) | 2.0 (0.0, 10.0) | 3.3 (0.0, 12.5) |
| Legumes (g/day) | 0.0 (0.0, 17.5) | 0.0 (0.0, 0.0) | 0.0 (0.0, 7.0) | 0.0 (0.0, 23.3) | 8.8 (0.0, 67.5) |
| Nuts (g/day) | 0.0 (0.0, 6.0) | 0.0 (0.0, 2.2) | 0.0 (0.0, 4.8) | 0.0 (0.0, 8.7) | 3.0 (0.0, 20.0) |
| Whole grains (g/day) | 211.8 (139.0, 301.5) | 169.0 (108.0, 236.5) | 201.2 (134.0, 288.8) | 232.4 (152.7, 318.5) | 268.0 (184.7, 351.7) |
| Fish (g/day) | 0.0 (0.0, 50.0) | 0.0 (0.0, 0.0) | 0.0 (0.0, 37.5) | 30.7 (0.0, 70.0) | 50.0 (0.0, 100.0) |
| Beef and lamb (g/day) | 0.0 (0.0, 60.0) | 40.0 (0.0, 75.0) | 0.0 (0.0, 60.0) | 0.0 (0.0, 30.0) | 0.0 (0.0, 0.0) |
| Pork (g/day) | 5.8 (0.0, 40.4) | 34.5 (7.7, 61.3) | 15.0 (0.0, 46.0) | 0.0 (0.0, 23.0) | 0.0 (0.0, 0.0) |
| Poultry(g/day) | 0.0 (0.0, 65.0) | 32.5 (0.0, 65.0) | 0.0 (0.0, 65.0) | 0.0 (0.0, 43.3) | 0.0 (0.0, 0.0) |
| Eggs (g/day) | 0.0 (0.0, 30.0) | 16.7 (0.0, 50.0) | 0.0 (0.0, 31.2) | 0.0 (0.0, 16.7) | 0.0 (0.0, 0.0) |
| Dairy (g/day) | 250.3 (160.5, 345.0) | 262.5 (170.0, 348.8) | 260.0 (170.0, 350.0) | 247.5 (160.0, 346.5) | 225.0 (133.3, 327.5) |
| Potatoes (g/day) | 87.5 (0.0, 175.0) | 126.7 (60.0, 180.0) | 90.0 (0.0, 175.0) | 60.0 (0.0, 140.0) | 30.0 (0.0, 90.0) |
| Added sugar (g/day) | 54.4 (35.9, 77.7) | 65.6 (46.8, 90.4) | 57.0 (38.6, 79.6) | 49.4 (32.4, 72.0) | 42.6 (26.9, 63.1) |

Data were presented as frequency (%), mean ± standard deviation or median (interquartile range).

Abbreviation: BMI, body mass index; eGFR, estimated glomerular filtration rate.

**Table S21. Baseline characteristics of the study participants according to categories of the Colizzi PHDI after imputation.**

| **Characteristics** | **Total** | **Categories of the Colizzi PHDI** | | | |
| --- | --- | --- | --- | --- | --- |
|  |  | **Q1** | **Q2** | **Q3** | **Q4** |
| No. participants | 193584 | 51003 | 49254 | 47375 | 45952 |
| Age (years) | 57.0 (50.0, 62.0) | 56.0 (48.0, 62.0) | 57.0 (50.0, 62.0) | 57.0 (50.0, 63.0) | 57.0 (50.0, 62.0) |
| Sex (male, %) | 86676 (44.8) | 26485 (51.9) | 22189 (45.1) | 20173 (42.6) | 17829 (38.8) |
| Ethnicity (%) |  |  |  |  |  |
| White | 185724 (95.9) | 48677 (95.4) | 47415 (96.3) | 45556 (96.2) | 44076 (95.9) |
| Asian | 2264 (1.2) | 907 (1.8) | 541 (1.1) | 471 (1.0) | 345 (0.8) |
| Black | 3099 (1.6) | 719 (1.4) | 700 (1.4) | 774 (1.6) | 906 (2.0) |
| Other | 2497 (1.3) | 700 (1.4) | 598 (1.2) | 574 (1.2) | 625 (1.4) |
| Education (%) |  |  |  |  |  |
| College or University degree | 90267 (46.6) | 20108 (39.4) | 21990 (44.6) | 23130 (48.8) | 25039 (54.5) |
| Other | 103317 (53.4) | 30895 (60.6) | 27264 (55.4) | 24245 (51.2) | 20913 (45.5) |
| Townsend deprivation index | -2.3 (-3.7, 0.0) | -2.2 (-3.7, 0.3) | -2.4 (-3.8, -0.1) | -2.4 (-3.8, -0.1) | -2.4 (-3.8, 0.0) |
| BMI (%, kg/m2) |  |  |  |  |  |
| Mean (SD) | 26.8 (4.6) | 27.5 (4.7) | 27.0 (4.6) | 26.6 (4.4) | 26.1 (4.4) |
| <18.5 | 1009 (0.5) | 204 (0.4) | 230 (0.5) | 249 (0.5) | 326 (0.7) |
| 18.5-24.9 | 72652 (37.5) | 15983 (31.3) | 17622 (35.8) | 18681 (39.4) | 20366 (44.3) |
| 25-29.9 | 80728 (41.7) | 22263 (43.7) | 20953 (42.5) | 19584 (41.3) | 17928 (39.0) |
| ≥30 | 39195 (20.2) | 12553 (24.6) | 10449 (21.2) | 8861 (18.7) | 7332 (16.0) |
| Smoking (n,%) |  |  |  |  |  |
| Never | 110174 (56.9) | 27447 (53.8) | 28231 (57.3) | 27418 (57.9) | 27078 (58.9) |
| Previous | 68319 (35.3) | 17765 (34.8) | 17202 (34.9) | 16856 (35.6) | 16496 (35.9) |
| Current | 15091 (7.8) | 5791 (11.4) | 3821 (7.8) | 3101 (6.5) | 2378 (5.2) |
| Alcohol intake (%) |  |  |  |  |  |
| Never | 11788 (6.1) | 3248 (6.4) | 2930 (5.9) | 2799 (5.9) | 2811 (6.1) |
| Special occasions only | 18668 (9.6) | 5048 (9.9) | 4894 (9.9) | 4438 (9.4) | 4288 (9.3) |
| 1-3 times/month | 21240 (11.0) | 5624 (11.0) | 5454 (11.1) | 5075 (10.7) | 5087 (11.1) |
| 1-2 times/week | 48329 (25.0) | 12554 (24.6) | 12484 (25.3) | 11898 (25.1) | 11393 (24.8) |
| 3-4 times/week | 49117 (25.4) | 12294 (24.1) | 12216 (24.8) | 12335 (26.0) | 12272 (26.7) |
| Daily or almost daily | 44442 (23.0) | 12235 (24.0) | 11276 (22.9) | 10830 (22.9) | 10101 (22.0) |
| Physical activity (%) |  |  |  |  |  |
| Low | 34684 (17.9) | 10650 (20.9) | 9065 (18.4) | 7948 (16.8) | 7021 (15.3) |
| Moderate | 82085 (42.4) | 21299 (41.8) | 20946 (42.5) | 20235 (42.7) | 19605 (42.7) |
| High | 76815 (39.7) | 19054 (37.4) | 19243 (39.1) | 19192 (40.5) | 19326 (42.1) |
| Cancer (%) | 20425 (10.6) | 5165 (10.1) | 5241 (10.6) | 5051 (10.7) | 4968 (10.8) |
| Hypertension (%) | 48211 (24.9) | 13511 (26.5) | 12534 (25.4) | 11556 (24.4) | 10610 (23.1) |
| Diabetes (%) | 7925 (4.1) | 2263 (4.4) | 2098 (4.3) | 1916 (4.0) | 1648 (3.6) |
| Cardiovascular disease (%) | 10717 (5.5) | 3135 (6.1) | 2790 (5.7) | 2541 (5.4) | 2251 (4.9) |
| eGFR (mL/min per 1.73 m^2^) | 93.5 (84.4, 100.4) | 93.4 (84.2, 100.7) | 93.2 (84.0, 100.2) | 93.4 (84.4, 100.2) | 94.0 (85.3, 100.7) |
| Total energy intake (kcal/day) | 1996.8 (1669.9, 2371.6) | 1944.5 (1583.0, 2359.6) | 1991.8 (1655.6, 2369.0) | 2007.0 (1693.9, 2367.4) | 2040.3 (1743.0, 2389.0) |
| Whole grains (g/day) | 211.8 (139.0, 301.5) | 163.0 (100.0, 269.2) | 204.0 (134.0, 299.5) | 224.0 (155.5, 305.0) | 244.2 (180.2, 319.2) |
| Potatoes (g/day) | 87.5 (0.0, 175.0) | 90.0 (0.0, 180.0) | 90.0 (0.0, 175.0) | 88.8 (14.6, 150.0) | 87.5 (43.8, 116.7) |
| Vegetables (g/day) | 170.0 (84.9, 280.8) | 90.0 (0.0, 187.9) | 156.6 (79.7, 266.5) | 191.8 (112.2, 298.9) | 235.7 (153.6, 338.8) |
| Fruits (g/day) | 180.0 (95.0, 293.0) | 100.0 (0.0, 200.0) | 174.0 (100.0, 284.0) | 210.0 (118.8, 315.5) | 237.0 (158.0, 345.0) |
| Dairy (g/day) | 250.3 (160.5, 345.0) | 210.0 (117.5, 330.0) | 253.7 (165.0, 350.0) | 261.7 (178.0, 347.5) | 270.0 (193.3, 348.8) |
| Red meat (g/day) | 46.0 (0.0, 94.0) | 76.0 (23.0, 120.0) | 47.7 (0.0, 98.0) | 40.0 (0.0, 79.5) | 23.0 (0.0, 60.0) |
| Poultry (g/day) | 0.0 (0.0, 65.0) | 0.0 (0.0, 50.0) | 0.0 (0.0, 65.0) | 0.0 (0.0, 65.0) | 32.5 (0.0, 50.0) |
| Eggs (g/day) | 0.0 (0.0, 30.0) | 0.0 (0.0, 25.0) | 0.0 (0.0, 30.0) | 0.0 (0.0, 33.3) | 12.5 (0.0, 30.0) |
| Fish (g/day) | 0.0 (0.0, 50.0) | 0.0 (0.0, 0.0) | 0.0 (0.0, 50.0) | 25.0 (0.0, 60.0) | 35.0 (7.5, 60.0) |
| Legumes | 0.0 (0.0, 11.2) | 0.0 (0.0, 0.0) | 0.0 (0.0, 0.0) | 0.0 (0.0, 17.5) | 8.8 (0.0, 35.0) |
| Soy food (g/day) | 0.0 (0.0, 0.0) | 0.0 (0.0, 0.0) | 0.0 (0.0, 0.0) | 0.0 (0.0, 0.0) | 0.0 (0.0, 0.0) |
| Nuts (g/day) | 0.0 (0.0, 6.0) | 0.0 (0.0, 0.0) | 0.0 (0.0, 4.0) | 0.0 (0.0, 8.7) | 3.8 (0.0, 16.6) |
| Unsaturated fat: saturated fat | 1.5 (1.2, 1.8) | 1.4 (1.1, 1.7) | 1.4 (1.2, 1.8) | 1.5 (1.2, 1.8) | 1.6 (1.3, 1.9) |
| Added sugar (g/day) | 54.4 (35.9, 77.7) | 58.6 (38.2, 85.6) | 54.7 (35.9, 78.3) | 53.2 (35.1, 75.2) | 51.5 (34.4, 72.1) |

Data were presented as frequency (%), mean ± standard deviation or median (interquartile range).

Abbreviation: BMI, body mass index; eGFR, estimated glomerular filtration rate.

**Table S22. Baseline characteristics of the study participants according to categories of the Knuppel PHDI after imputation.**

| **Characteristics** | **Total** | **Categories of the Knuppel PHDI** | | | |
| --- | --- | --- | --- | --- | --- |
|  |  | **≤8** | **9** | **10** | **≥11** |
| No. participants | 193584 | 53866 | 56266 | 49408 | 34044 |
| Age (years) | 57.0 (50.0, 62.0) | 56.0 (49.0, 62.0) | 57.0 (50.0, 62.0) | 57.0 (50.0, 62.0) | 57.0 (50.0, 62.0) |
| Sex (male, %) | 86676 (44.8) | 31030 (57.6) | 26759 (47.6) | 19068 (38.6) | 9819 (28.8) |
| Ethnicity (%) |  |  |  |  |  |
| White | 185724 (95.9) | 51994 (96.5) | 54157 (96.3) | 47300 (95.7) | 32273 (94.8) |
| Asian | 2264 (1.2) | 610 (1.1) | 646 (1.1) | 580 (1.2) | 428 (1.3) |
| Black | 3099 (1.6) | 636 (1.2) | 787 (1.4) | 839 (1.7) | 837 (2.5) |
| Other | 2497 (1.3) | 626 (1.2) | 676 (1.2) | 689 (1.4) | 506 (1.5) |
| Education (%) |  |  |  |  |  |
| College or University degree | 90267 (46.6) | 23842 (44.3) | 25828 (45.9) | 23475 (47.5) | 17122 (50.3) |
| Other | 103317 (53.4) | 30024 (55.7) | 30438 (54.1) | 25933 (52.5) | 16922 (49.7) |
| Townsend deprivation index | -2.3 (-3.7, 0.0) | -2.3 (-3.7, 0.0) | -2.4 (-3.8, 0.0) | -2.4 (-3.8, 0.0) | -2.2 (-3.7, 0.2) |
| BMI (%, kg/m2) |  |  |  |  |  |
| Mean (SD) | 26.8 (4.6) | 27.2 (4.6) | 26.9 (4.5) | 26.7 (4.6) | 26.3 (4.5) |
| <18.5 | 1009 (0.5) | 212 (0.4) | 250 (0.4) | 281 (0.6) | 266 (0.8) |
| 18.5-24.9 | 72652 (37.5) | 18116 (33.6) | 20557 (36.5) | 19269 (39.0) | 14710 (43.2) |
| 25-29.9 | 80728 (41.7) | 23437 (43.5) | 23919 (42.5) | 20155 (40.8) | 13217 (38.8) |
| ≥30 | 39195 (20.2) | 12101 (22.5) | 11540 (20.5) | 9703 (19.6) | 5851 (17.2) |
| Smoking (n,%) |  |  |  |  |  |
| Never | 110174 (56.9) | 29873 (55.5) | 32271 (57.4) | 28399 (57.5) | 19631 (57.7) |
| Previous | 68319 (35.3) | 19021 (35.3) | 19506 (34.7) | 17552 (35.5) | 12240 (36.0) |
| Current | 15091 (7.8) | 4972 (9.2) | 4489 (8.0) | 3457 (7.0) | 2173 (6.4) |
| Alcohol intake (%) |  |  |  |  |  |
| Never | 11788 (6.1) | 2984 (5.5) | 3223 (5.7) | 3094 (6.3) | 2487 (7.3) |
| Special occasions only | 18668 (9.6) | 4865 (9.0) | 5228 (9.3) | 4845 (9.8) | 3730 (11.0) |
| 1-3 times/month | 21240 (11.0) | 5799 (10.8) | 6101 (10.8) | 5472 (11.1) | 3868 (11.4) |
| 1-2 times/week | 48329 (25.0) | 13422 (24.9) | 14180 (25.2) | 12419 (25.1) | 8308 (24.4) |
| 3-4 times/week | 49117 (25.4) | 13706 (25.4) | 14439 (25.7) | 12532 (25.4) | 8440 (24.8) |
| Daily or almost daily | 44442 (23.0) | 13090 (24.3) | 13095 (23.3) | 11046 (22.4) | 7211 (21.2) |
| Physical activity (%) |  |  |  |  |  |
| Low | 34684 (17.9) | 10671 (19.8) | 10334 (18.4) | 8437 (17.1) | 5242 (15.4) |
| Moderate | 82085 (42.4) | 22788 (42.3) | 24034 (42.7) | 20926 (42.4) | 14337 (42.1) |
| High | 76815 (39.7) | 20407 (37.9) | 21898 (38.9) | 20045 (40.6) | 14465 (42.5) |
| Cancer (%) | 20425 (10.6) | 5164 (9.6) | 5801 (10.3) | 5507 (11.1) | 3953 (11.6) |
| Hypertension (%) | 48211 (24.9) | 13991 (26.0) | 14176 (25.2) | 12173 (24.6) | 7871 (23.1) |
| Diabetes (%) | 7925 (4.1) | 2207 (4.1) | 2269 (4.0) | 2071 (4.2) | 1378 (4.0) |
| Cardiovascular disease (%) | 10717 (5.5) | 3183 (5.9) | 3298 (5.9) | 2670 (5.4) | 1566 (4.6) |
| eGFR (mL/min per 1.73 m^2^) | 93.5 (84.4, 100.4) | 93.3 (84.2, 100.5) | 93.3 (84.2, 100.3) | 93.6 (84.6, 100.4) | 94.0 (85.1, 100.7) |
| Total energy intake (kcal/day) | 1996.8 (1669.9, 2371.6) | 2203.3 (1885.5, 2580.1) | 2034.3 (1725.8, 2390.9) | 1908.7 (1604.4, 2260.9) | 1728.3 (1424.8, 2070.8) |
| Whole grains (g/day) | 211.8 (139.0, 301.5) | 254.5 (166.0, 325.6) | 219.5 (144.0, 308.3) | 194.5 (131.0, 286.0) | 172.5 (115.0, 229.5) |
| Potatoes (g/day) | 87.5 (0.0, 175.0) | 126.7 (45.0, 180.0) | 90.0 (0.0, 175.0) | 66.7 (0.0, 143.8) | 43.8 (0.0, 90.0) |
| Vegetables (g/day) | 170.0 (84.9, 280.8) | 126.7 (66.0, 186.0) | 153.1 (75.5, 253.7) | 209.0 (95.0, 311.0) | 261.0 (168.8, 372.5) |
| Fruits (g/day) | 180.0 (95.0, 293.0) | 98.5 (20.0, 214.5) | 173.2 (96.0, 280.0) | 212.0 (121.0, 320.0) | 243.3 (159.7, 362.5) |
| Dairy (g/day) | 250.3 (160.5, 345.0) | 260.0 (170.0, 362.5) | 254.7 (165.0, 347.0) | 248.1 (160.0, 338.3) | 240.0 (145.0, 330.0) |
| Red meat (g/day) | 46.0 (0.0, 94.0) | 60.0 (30.7, 115.3) | 55.3 (0.0, 101.5) | 32.2 (0.0, 87.7) | 0.0 (0.0, 53.0) |
| Poultry (g/day) | 0.0 (0.0, 65.0) | 32.5 (0.0, 65.0) | 0.0 (0.0, 65.0) | 0.0 (0.0, 43.3) | 0.0 (0.0, 32.5) |
| Eggs (g/day) | 0.0 (0.0, 30.0) | 16.7 (0.0, 50.0) | 0.0 (0.0, 30.0) | 0.0 (0.0, 16.7) | 0.0 (0.0, 0.0) |
| Fish (g/day) | 0.0 (0.0, 50.0) | 0.0 (0.0, 50.0) | 0.0 (0.0, 50.0) | 0.0 (0.0, 50.0) | 15.3 (0.0, 60.0) |
| Legumes | 0.0 (0.0, 11.2) | 0.0 (0.0, 17.5) | 0.0 (0.0, 8.4) | 0.0 (0.0, 7.0) | 0.0 (0.0, 8.8) |
| Soy food (g/day) | 0.0 (0.0, 0.0) | 0.0 (0.0, 0.0) | 0.0 (0.0, 0.0) | 0.0 (0.0, 0.0) | 0.0 (0.0, 0.0) |
| Nuts (g/day) | 0.0 (0.0, 6.0) | 0.0 (0.0, 3.0) | 0.0 (0.0, 5.0) | 0.0 (0.0, 7.0) | 1.5 (0.0, 20.0) |
| Unsaturated fat: saturated fat | 1.5 (1.2, 1.8) | 1.4 (1.2, 1.7) | 1.4 (1.2, 1.7) | 1.5 (1.2, 1.8) | 1.6 (1.3, 2.1) |
| Added sugar (g/day) | 54.4 (35.9, 77.7) | 64.1 (46.2, 88.0) | 57.6 (40.5, 80.3) | 50.8 (32.6, 72.9) | 34.1 (22.1, 59.2) |

Data were presented as frequency (%), mean ± standard deviation or median (interquartile range).

Abbreviation: BMI, body mass index; eGFR, estimated glomerular filtration rate.

**Table S23. Associations between the different PHDI and risks of CKD after imputation.**

|  | **Cases/Total** | **Model 1^a^** | | **Model 2^b^** | | **Model 3^b^** | |
| --- | --- | --- | --- | --- | --- | --- | --- |
|  |  | **HR (95% CI)** | ***P value*** | **HR (95% CI)** | ***P value*** | **HR (95% CI)** | ***P value*** |
| Stubbendorff PHDI |  |  |  |  |  |  |  |
| ≤18 | 2321/41074 | REF |  | REF |  | REF |  |
| 19-22 | 3598/66701 | 0.918 (0.871-0.967) | 0.001 | 0.925 (0.878-0.975) | 0.004 | 0.955 (0.906-1.006) | 0.084 |
| 23-26 | 2752/56483 | 0.830 (0.785-0.877) | <0.001 | 0.847 (0.801-0.895) | <0.001 | 0.909 (0.859-0.961) | 0.001 |
| ≥27 | 1193/29326 | 0.702 (0.654-0.753) | <0.001 | 0.726 (0.677-0.780) | <0.001 | 0.828 (0.771-0.889) | <0.001 |
| *P* for trend |  | <0.001 |  | <0.001 |  | <0.001 |  |
| 1-point increment in diet index | 9864/193584 | 0.974 (0.969-0.979) | <0.001 | 0.977 (0.972-0.981) | <0.001 | 0.987 (0.982-0.991) | <0.001 |
| Colizzi PHDI |  |  |  |  |  |  |  |
| Q1 | 2944/51003 | REF |  | REF |  | REF |  |
| Q2 | 2604/49254 | 0.873 (0.828-0.921) | <0.001 | 0.895 (0.849-0.944) | <0.001 | 0.919 (0.872-0.970) | 0.002 |
| Q3 | 2326/47375 | 0.797 (0.755-0.842) | <0.001 | 0.835 (0.790-0.881) | <0.001 | 0.883 (0.835-0.933) | <0.001 |
| Q4 | 1990/45952 | 0.707 (0.668-0.749) | <0.001 | 0.756 (0.714-0.801) | <0.001 | 0.833 (0.786-0.883) | <0.001 |
| *P* for trend |  | <0.001 |  | <0.001 |  | <0.001 |  |
| 10-point increment in diet index | 9864/193584 | 0.913 (0.900-0.926) | <0.001 | 0.931 (0.918-0.944) | <0.001 | 0.955 (0.941-0.968) | <0.001 |
| Knuppel PHDI |  |  |  |  |  |  |  |
| ≤9 | 2907/53866 | REF |  | REF |  | REF |  |
| 10 | 2909/56266 | 0.939 (0.892-0.989) | 0.017 | 0.944 (0.897-0.994) | 0.030 | 0.963 (0.914-1.014) | 0.154 |
| 11 | 2434/49408 | 0.896 (0.849-0.946) | <0.001 | 0.900 (0.852-0.950) | <0.001 | 0.939 (0.888-0.992) | 0.026 |
| ≥12 | 1614/34044 | 0.875 (0.822-0.931) | <0.001 | 0.891 (0.837-0.948) | <0.001 | 0.950 (0.891-1.014) | 0.122 |
| *P* for trend |  | <0.001 |  | <0.001 |  | 0.048 |  |
| 1-point increment in diet index | 9864/193584 | 0.960 (0.946-0.975) | <0.001 | 0.964 (0.950-0.979) | <0.001 | 0.981 (0.965-0.997) | 0.017 |

^a^Model 1 was adjusted for age, sex, ethnicity;

^b^Model 2 was adjusted for model 1 plus Townsend deprivation index, education, hypertension, diabetes, cardiovascular disease, cancer;

^c^Model 3 was adjusted for model 2 plus BMI, eGFR, smoking status, alcohol intake, physical activity, total energy intake.

**Reference**

1. Malloy EJ, Spiegelman D, Eisen EA. Comparing measures of model selection for penalized splines in Cox models. *Comput Stat Data Anal*. 2009;53(7):2605-2616. doi:10.1016/j.csda.2008.12.008

2. Eisen EA, Agalliu I, Thurston SW, Coull BA, Checkoway H. Smoothing in occupational cohort studies: an illustration based on penalised splines. *Occup Environ Med*. 2004;61(10):854-860. doi:10.1136/oem.2004.013136

3. White IR, Royston P, Wood AM. Multiple imputation using chained equations: Issues and guidance for practice. *Stat Med*. 2011;30(4):377-399. doi:10.1002/sim.4067

4. Piernas C, Perez-Cornago A, Gao M, et al. Describing a new food group classification system for UK biobank: analysis of food groups and sources of macro- and micronutrients in 208,200 participants. *Eur J Nutr*. 2021;60(5):2879-2890. doi:10.1007/s00394-021-02535-x

5. Perez-Cornago A, Pollard Z, Young H, et al. Description of the updated nutrition calculation of the Oxford WebQ questionnaire and comparison with the previous version among 207,144 participants in UK Biobank. *Eur J Nutr*. 2021;60(7):4019-4030. doi:10.1007/s00394-021-02558-4

6. Stubbendorff A, Sonestedt E, Ramne S, Drake I, Hallström E, Ericson U. Development of an EAT-Lancet index and its relation to mortality in a Swedish population. *Am J Clin Nutr*. 2022;115(3):705-716. doi:10.1093/ajcn/nqab369

7. Willett W, Rockström J, Loken B, et al. Food in the Anthropocene: the EAT-Lancet Commission on healthy diets from sustainable food systems. *Lancet*. 2019;393(10170):447-492. doi:10.1016/S0140-6736(18)31788-4

8. Colizzi C, Harbers MC, Vellinga RE, et al. Adherence to the EAT-Lancet Healthy Reference Diet in Relation to Risk of Cardiovascular Events and Environmental Impact: Results From the EPIC-NL Cohort. *J Am Heart Assoc*. 2023;12(8):e026318. doi:10.1161/JAHA.122.026318

9. Knuppel A, Papier K, Key TJ, Travis RC. EAT-Lancet score and major health outcomes: the EPIC-Oxford study. *Lancet*. 2019;394(10194):213-214. doi:10.1016/S0140-6736(19)31236-X

10. Wuttke M, Li Y, Li M, et al. A catalog of genetic loci associated with kidney function from analyses of a million individuals. *Nat Genet*. 2019;51(6):957-972. doi:10.1038/s41588-019-0407-x

11. Yu Z, Jin J, Tin A, et al. Polygenic Risk Scores for Kidney Function and Their Associations with Circulating Proteome, and Incident Kidney Diseases. *J Am Soc Nephrol*. 2021;32(12):3161-3173. doi:10.1681/ASN.2020111599
